# The trouble with free-water elimination using single-shell diffusion MRI data: A case study in ageing

**DOI:** 10.1162/imag_a_00252

**Published:** 2024-08-01

**Authors:** Marta M. Correia, Rafael Neto Henriques, Marc Golub, Stefan Winzeck, Rita G. Nunes

**Affiliations:** MRC Cognition and Brain Sciences Unit, University of Cambridge, Cambridge, United Kingdom; Champalimaud Research, Champalimaud Centre for the Unknown, Lisbon, Portugal; Institute for Systems and Robotics—Lisboa and Department of Bioengineering, Instituto Superior Técnico, Universidade de Lisboa, Lisbon, Portugal; Division of Anaesthesia, Department of Medicine, University of Cambridge, Cambridge, United Kingdom

**Keywords:** diffusion MRI, free-water elimination, ageing

## Abstract

Free-water elimination (FWE) modelling for diffusion tensor imaging (DTI) can be used to estimate the free-water (FW) volume fraction, as well as FW-compensated DTI parameters. Single-shell (SS) diffusion magnetic resonance imaging (MRI) acquisitions are more common in clinical cohorts due to time constraints, but the FWE-DTI model is a two-compartment model, hence only well posed for multi-shell (MS) data. A regularised gradient descent (RGD) method is often applied to SS datasets and has been used to study healthy ageing, Alzheimer’s and Parkinson’s disease, amongst others, largely ignoring the methodological limitations of this approach. In this study, we compared the performance of RGD fitting with SS data, to a non-linear least squares (NLS) fitting applied to MS data, using simulations and data from 620 participants aged 18 to 88 years. Consistent with previous studies, our simulations show that RGD fitting using SS data flattens the relationship between mean diffusivity (MD) estimates and their ground truth values, and introduces an artificial positive correlation between fractional anisotropy (FA) estimates and the underlying tissue ground truth MD. Neither of these biases were observed when NLS fitting was applied to MS data. In human data, a smaller number of significant voxels with positive correlations between MD and age were observed when the RGD SS algorithm was used, which is consistent with the flattening of MD profiles observed in simulations. FW-compensated FA maps produced strikingly different results depending on the method employed: the maps obtained with RGD SS identified some brain areas with a strong positive association with age, while no such positive correlations were found with MS NLS. While similar positive correlations between age and FW-compensated FA maps obtained with SS RGD have been reported, these results are only replicated when the RGD SS was used, suggesting that this apparent FA increase was likely an artefact introduced by inappropriate modelling using SS data. Our study, therefore, suggests that previous findings reported in the literature using the RGD approach should be interpreted with extreme care.

## 1 Introduction

Diffusion tensor imaging (DTI) is a magnetic resonance imaging (MRI) modality which enables the non-invasive mapping of the trajectories of water molecules in vivo in humans (Basser et al., 1994;Pierpaoli et al., 1996). Since it was first described in the 1990s, DTI has gained immense popularity amongst clinical research communities, and it has been particularly successful in the study of brain white matter. The applications of DTI span a wide range of conditions, including stroke, Alzheimer’s disease and dementia, movement disorders, traumatic brain injury (TBI), schizophrenia, autism spectrum disorders (ASD), healthy ageing and early development (Chanraud et al., 2010;Ciccarelli et al., 2008;Douglas et al., 2015;Feldman et al., 2010;Madden et al., 2012;Oishi et al., 2011;Sullivan & Pfefferbaum, 2006;Sundgren et al., 2004;Tae et al., 2018;Y. Zhang & Burock, 2020).

Despite its many successes, DTI also suffers from several limitations and methodological constraints (Soares et al., 2013). One such issue is the partial volume contamination from neighbouring cerebral spinal fluid (CSF), also commonly referred to as free-water (FW) contamination. Because the DTI model assumes the signal comes from a single compartment, even the smallest contamination from FW will result in biased tensor estimates and corresponding metrics (Metzler-Baddeley et al., 2012). This limitation can be addressed by introducing an FW compartment into the DTI model. This free-water elimination (FWE) approach was first introduced byPierpaoli and Jones (2004). Besides estimating the volume fraction of FW (FW index), this model can also estimate FW-compensated DTI metrics, including mean diffusivity (MD) and fractional anisotropy (FA).

The FWE-DTI model is a two-compartment model, hence only well posed for data which includes two or more non-zero b-values (multi-shell (MS) data) (Bergmann et al., 2020;Hoy et al., 2014). However, single-shell (SS) diffusion MRI acquisitions (with a single non-zero b-value) are more common in clinical cohorts due to time constraints. To overcome this limitation,Pasternak et al. (2009)proposed a spatially regularised gradient descent (RGD) algorithm to fit the FWE-DTI model to SS data, which is highly dependent on the initialisation method. This regularised approach has been used to study healthy ageing (Chad et al., 2018), Alzheimer’s (Hoy et al., 2017), Parkinson’s disease (Planetta et al., 2016), and traumatic brain injury (Vijayakumari et al., 2021), amongst others. However, none of these applied studies took into consideration the methodological limitations of RGD approaches when interpreting their findings.

Golub et al. (2021)performed a systematic evaluation of the biases resulting from RGD algorithms for FWE-DTI fitting, using both simulations and human diffusion MRI data. In summary, they found that while the estimates obtained from RGD fitting seemed biologically plausible, this technique was unable to differentiate between changes in the FW index and changes in the MD of the tissue. Their results suggest that diffusion metrics obtained from RGD methods applied to SS data lack specificity, and could potentially lead to erroneous interpretations when applied to human datasets.

Age-related brain degeneration results in increased CSF volume (Yamada et al., 2023), making the study of white matter changes with age particularly vulnerable to FW contamination biases. To date, only a few studies have investigated the impact of FWE in healthy ageing.Metzler-Baddeley et al. (2012)applied FWE-DTI using RGD fitting to a sample of 39 healthy adults aged 53 to 93 years and found that strong positive correlations between uncorrected MD and age disappeared in the fornix after correction for FW contamination. The strength of the correlation with FA remained largely unchanged after correction. The authors hence concluded that the correlations between age and uncorrected MD were driven by CSF contamination, and that FA estimates were more robust to FW contamination than diffusivity-based metrics. These observations were partially reproduced inChad et al. (2018), using a larger sample of 212 participants, aged 39 to 91 years. In this study, also using RGD fitting, the authors found much reduced correlations between MD and age after FW correction, but they also reported some positive correlations between corrected FA and age, contrasting the prevalence of FA decline with age in the literature (e.g.,Sullivan et al., 2001). The latter were interpreted as related to selective degeneration of non-dominant tracts at fibre crossings. A more recent study byPieciak et al. (2023)used a spherical means-based FW estimation approach applied to MS data to examine 287 healthy adults aged 25 to 94 years. A flattening effect of the corrected MD and FA age profiles relative to standard DTI metrics was reported, and the positive FA correlations identified byChad et al. (2018)were not reproduced. Finally,Nemmi et al. (2022)compared the performance of SS RGD model fitting with MS NLS in the context of brain-age estimation models, using a sample of 89 males, aged 20 to 85 years. The authors found that MS NLS modelling significantly increased the prediction accuracy of the brain-age models tested, when compared with SS RGD fitting. However, they made no attempts to understand the underlying causes for the discrepancies observed, citing only that SS RGD is sensitive to initialisation, which could lead to inaccurate reconstruction.

The goal of the present study was to build on the findings ofGolub et al. (2021), by comparing the performance of RGD fitting with SS data, to a non-linear least squares (NLS) fitting applied to MS data, in the context of healthy ageing. Our aim was to determine whether both methods lead to comparable research findings, or whether the differences between them could explain some of the discrepancies in published studies. For this purpose, we used a sample of 620 datasets from the Cam-CAN project (Shafto et al., 2014).

## 2 Methods

### 2.1 Simulations

Four simulation experiments were performed to provide an expectation of the accuracy of FW estimates obtained from the SS and MS FWE-DTI fitting approaches for different acquisitions and considering different effects of water diffusion in biological systems. The code used for the simulations is available here (https://github.com/RafaelNH/fweDTI_SSvsMS).

For simulation experiments 1 and 2, synthetic signals were produced using a forward model that matches the assumptions of the FW DTI model (Pasternak et al., 2009;Pierpaoli & Jones, 2004). The goal of these was to assess the accuracy of the fitting routines independently of possible discrepancies between the underlying model and real biological tissues, while differing in the set of sampled b-values. Similar to the work byGolub et al. (2021), simulations were repeated for different ground truth FW volume fractions (sampled from 0 to 0.7), for different tissue mean diffusivities ($MD_{tis}$, sampled from 0.2 to 1.1 μm^2^/ms), and for a tissue fractional anisotropy$\left(FA_{tis}\right)$fixed to 0.7.

For simulation experiments 3 and 4, tissue non-Gaussian effects were included by independently modelling signal contributions from intra- and extra-cellular tissue compartments, in addition to an FW compartment. The diffusivities and volume fractions of the intra- and extra-cellular compartments were computed according to the conversion between the Neurite Orientation and Dispersion Imaging and DTI (Edwards et al., 2017;Jespersen et al., 2012;H. Zhang et al., 2012), to allow the definition of all model parameters according to the desired$MD_{tis}$and$FA_{tis}$values.

For all experiments, simulations were repeated for 10,000 noisy instances (Rician noise, SNR = 30) and performed along 30 directions for b-values of 300 and 1000 s/mm^2^(experiments 1 and 3) and b-values of 1000 and 2000 s/mm^2^(experiments 2 and 4) in addition to three b-value= 0 s/mm^2^signals. These b-value pairs are chosen to match the b-values collected for human data.

For all above simulations, single-shell (SS) FWE-DTI fits were performed for the initialisation method based on a tissue’s MD prior set to 0.6 μm^2^/ms (Golub et al., 2021) and without performing the Laplace–Beltrami spatial regularisation, as this is incompatible with single voxel simulates. However, results from these single voxel simulations are expected to be representative of the (SS) FWE-DTI fits with spatial regularisation obtained from the human data, becauseGolub et al. (2021)showed that the accuracy of this technique is determined by its initialisation routine. More details on the simulation generation and processing are reported inSupplementary Material, Appendix A. This Supplementary Material also included multi-voxel simulation to showcase the generalisability of our simulation results to SS FWE-DTI fittings including spatial regularisation (c.f.Supplementary Fig. 1).

### 2.2 Participants

#### 2.2.1 Cam-CAN dataset

Our sample for the ageing analysis included 626 healthy participants with a complete diffusion MRI scan (age range: 18–88 years, 317 females) from the Cam-CAN dataset (https://www.cam-can.org). Approval for the Cam-CAN study was granted by the Research Ethics Committee of Cambridgeshire 2 (now known as East of England—Cambridge Central), and participants provided written, informed consent prior to their involvement. These participants were selected from 2681 participants interviewed in their homes and met all the inclusion criteria as set out inShafto et al. (2014). After data quality control (see below), a final sample of 620 participants (age range: 18–88 years, 315 females) was included in subsequent analyses.

#### 2.2.2 Extra dataset with lower b-values

The diffusion MRI sequence used to collect the Cam-CAN data includes two b-values (see below for details), which makes it suitable for FWE-DTI model fitting using the MS NLS. However, the highest of those b-values is 2000 s/mm^2^, which means non-Gaussian diffusion effects are likely to introduce biases in the diffusion tensor estimates (Collier et al., 2018;Jensen et al., 2005). To assess the impact of these non-Gaussian effects, three additional healthy controls were recruited for this study (age range: 39–42 years, 2 females). These extra controls were scanned using the same sequence used for the Cam-CAN dataset, but an additional b-value of 300 s/mm^2^shell was also collected (see below for details). Local Ethical Committee approval and written informed consent were obtained.

### 2.3 MRI data acquisition

Diffusion and T1-weighted MRI data were acquired for all Cam-CAN participants on a 3T Siemens Tim TRIO scanner at the MRC Cognition and Brain Sciences Unit, using a 32-channel head coil. Diffusion MRI data were acquired with a twice refocused spin echo (TRSE) sequence (Reese et al., 2003). Diffusion sensitising gradients were applied along 30 non-collinear directions for each of the two b-values (b = 1000 and b = 2000 s/mm^2^), together with three acquisitions without diffusion weighting (b = 0 s/mm^2^). The remaining imaging parameters were TR = 9100 ms, TE = 104 ms, matrix = 96 × 96, field of view (FoV) = 192 × 192 mm^2^, slice thickness = 2 mm without gap, interleaved slice acquisition order, partial Fourier of 7/8, and acceleration factor of 2 using GRAPPA with 36 reference lines. A high-resolution 3D T1-weighted MPRAGE image was also acquired (TR = 2300 ms, TE = 2.98 ms, FoV = 256 × 240 mm^2^, matrix = 256 × 256, slice thickness = 1 mm). More information about the MRI acquisition is reported inTaylor et al. (2017). The raw data in BIDS format are available upon request fromhttps://Cam-CAN-archive.mrc-cbu.cam.ac.uk/dataaccess/.

The three extra healthy volunteers were scanned using a 3T Siemens PRISMA Fit scanner at the MRC Cognition and Brain Sciences Unit and a 32-channel head coil. All acquisition parameters were the same as described above for the main Cam-CAN dataset, but an additional b-value of 300 s/mm^2^was also collected for the same set of 30 directions. It should be noted that it was not possible to use the exact same MRI scanner for data collection because the 3T TRIO used for acquisition of all Cam-CAN datasets was upgraded to a PRISMA Fit in the meantime.

### 2.4 Data pre-processing and model fitting

All Cam-CAN data were processed with a common pipeline that has been described in detail inWinzeck (2021). In short, diffusion MRI data were corrected for noise and Gibbs ringing using tools implemented in DIPY (https://dipy.org/), eddy current distortions, and head movement were corrected using eddy in FSL (https://fsl.fmrib.ox.ac.uk/fsl/fslwiki/). After these pre-processing steps, six datasets were excluded from further analysis: two due to corrupted diffusion-weighted images, resembling “salt and pepper,” and four due to excessive motion artefacts. Demographic information about the 320 participants included in subsequent analyses is provided inTable 1. Correction for B0 field inhomogeneities was not applied because reverse phase-encode direction data were not available. DTI fitting was performed by excluding the b = 2000 s/mm^2^data (DTI SS), using weighted linear least squares fitting in FSL, and mean diffusivity (MD) and fractional anisotropy (FA) maps were generated. FWE-DTI modelling was performed in two ways: firstly, the full dataset was used to estimate FW index and FWE-DTI parameters using the MS NLS approach (Hoy et al., 2014;Neto Henriques et al., 2017); secondly, the b = 2000 s/mm^2^data were excluded and the remaining data were used to estimate FW index and FWE-DTI parameters using an RGD approach for SS data (Golub et al., 2021;Pasternak et al., 2009) with the initialisation method based on the tissue’s MD, as described inGolub et al. (2021). SS and MS FWE-DTI fitting were performed using the fitting procedures implemented, respectively, byGolub et al. (2021)andNeto Henriques et al. (2017)in DIPY. In both cases, a threshold of FW index <0.7 was applied before diffusion tensor fitting to exclude voxels with excessive FW contamination (Neto Henriques et al., 2017). As a result of the different fitting methods described above, for each study volunteer, we generated eight whole brain maps of diffusion metrics: dti_FA_SS, dti_MD_SS, fwe_FA_SS, fwe_MD_SS, fwe_FW_SS, fwe_FA_MS, fwe_MD_MS, and fwe_FW_MS.

**Table 1.** Demographic information for the subset of Cam-CAN participants included in this study.

	Number of participants	Mean age	Standard deviation	Min age	Max age
Female	315	54.11	18.88	18	88
Male	305	54.90	17.93	18	87
All	620	54.50	18.41	18	88

For the three datasets with an extra b = 300 s/mm^2^shell, the b = 300 s/mm^2^data were first excluded and the same pipeline was employed, resulting in the same eight metrics as described above for the Cam-CAN data. In addition, FWE-DTI using the MS NLS fitting was also applied to the b-value pair b = 300, 1000 s/mm^2^to obtain FWE-DTI metrics with minimised tissue non-Gaussian diffusion effects (Hoy et al., 2014). This resulted in three additional brain maps of diffusion metrics: fwe_FA_GS, fwe_MD_GS, and fwe_FW_GS. Because these maps were obtained with the well-posed NLS fitting model and are expected to be less affected by non-Gaussian diffusion effects due to the low b-values used, we shall refer to them as “Gold Standard” (GS) for the remainder of this manuscript.

### 2.5 TBSS group analyses

The standard TBSS procedures (Smith et al., 2006) were applied to the dti_FA_SS data for the Cam-CAN dataset, using FSL. The tbss_non_FA script was then applied to the other seven maps (dti_MD_SS, fwe_FA_SS, fwe_MD_SS, fwe_FW_SS, fwe_FA_MS, fwe_MD_MS, and fwe_FW_MS) to generate skeletonised data for all eight diffusion metrics. FSL’s randomise with 5000 permutations was used to identify which skeleton voxels correlate positively or negatively with age for each of the eight metrics, using Threshold-Free Cluster Enhancement (TFCE) for correction for multiple comparisons. Sex was also included as a covariate. Significant correlation between age and diffusion metrics was defined by a corrected p-value <0.05. The cluster volume (number of significant voxels) was calculated for each metric, for both positive and negative correlation with age. Only the Cam-CAN data were included in these analyses.

### 2.6 ROI analysis

The JHU white matter labels atlas was used for ROI-based analyses (JHU ROIs). For more information, seehttps://fsl.fmrib.ox.ac.uk/fsl/fslwiki/Atlases/, andMori and Crain (2005).

The 48 JHU ROIs were back projected to the diffusion MRI space for each participant. First, each individual dti_FA_SS map was spatially normalised to the JHU FA template provided by FSL using antsRegistration (http://stnava.github.io/ANTs/). Subsequently, the inverse transformation was applied to the JHU ROIs using nearest-neighbours interpolation, and the means of each diffusion metric within each ROI were extracted in subject-specific space. The ROIs corresponding to the same brain region, but different hemispheres, were merged, resulting in a total of 27 ROIs per subject.

For the three extra human participants with data acquired with lower b-values, the mean values for fwe_MD_SS and fwe_MD_MS were plotted against the “gold standard” MD estimates (fwe_MD_GS) for each JHU ROI, and the same plots were replicated for FA and FW index. This will allow us to evaluate any biases introduced by the fitting method, as well as the impact of non-Gaussian diffusion effects due to the high b-value = 2000 s/mm^2^used for the FWE model estimation, in a small set of datasets, which are independent of the main Cam-CAN data.

For the Cam-CAN dataset, we fitted a linear model for each ROI and each metric, with age as an explanatory variable and sex as a nuisance covariate. Analysis of variance (ANOVA) was used to assess the significance of the regression in each case. To further explore the impact of the choice of FWE fitting algorithm on the FA estimates, analysis of covariance (ANCOVA) was also performed within each ROI, including age and fitting method as main factors, as well as the interaction between them. To account for multiple comparisons, all results were corrected for false discovery rate (FDR) using the Benjamini–Hochberg method (Haynes, 2013), with an FDR rate of 0.05. While other studies have shown quadratic relationships between some diffusion metrics and age (e.g.,Neto Henriques et al., 2023), we chose linear models for the present study to facilitate the comparison with previous studies that reported positive linear correlations between FA and age. Note that the goal of the present study was not to further develop the understanding of the ageing process itself by finding the model which best explains the variance in the data with age, instead our main focus was to compare the performance of two FWE-DTI fitting algorithms.

## 3 Results

### 3.1 Simulations

**Fig. 1.**
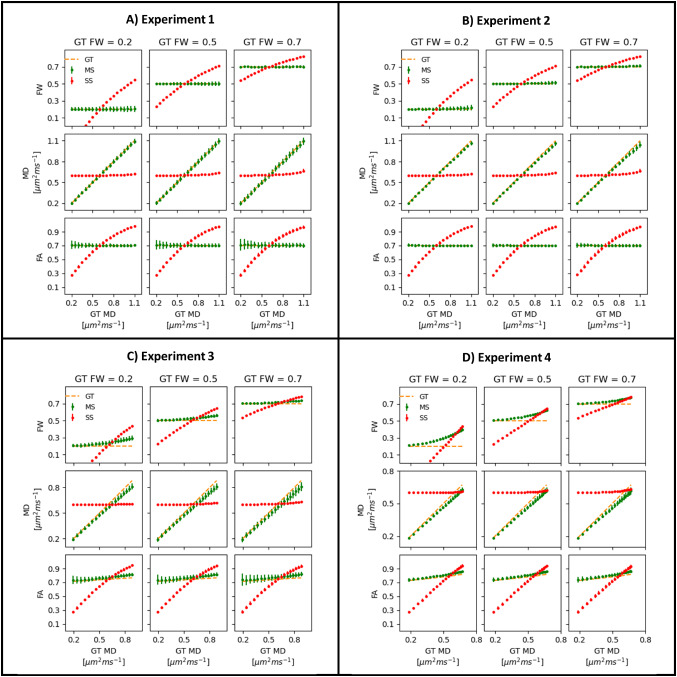
Single-shell (SS) and multi-shell (MS) FWE-DTI estimated for four simulation experiments considering different acquisition parameters and distinct effects of water diffusion in biological systems: (A) synthetic signals for b = 300, 1000 s/mm^2^ignoring non-Gaussian diffusion effects; (B) synthetic signals for b = 1000, 2000 s/mm^2^ignoring non-Gaussian diffusion effects; (C) synthetic signals for b = 300, 1000 s/mm^2^and considering non-Gaussian diffusion effects; (D) synthetic signals for b = 1000, 2000 s/mm^2^considering non-Gaussian diffusion effects. For each panel, FW, MD, and FA estimates are plotted as a function of the ground truth MD (1st, 2^nd^, and 3rd rows of plots, respectively) for different ground truth free-water contaminations (FW = 0.2, 0.5, 07 for columns of plots, respectively).

Figure 1Ashows the plots obtained for Experiment 1 (single-voxel simulations matching the assumptions of the FWE-DTI model for b = 300 and 1000 s/mm^2^). The parameter estimates obtained with NLS fitting (MS) generally follow the simulated ground truth (GT), while the results obtained with RGD fitting (SS) show clear biases, in particular the estimated FA value increases with the underlying MD of the tissue.Figure 1Bshows the corresponding plots for the b-value pair b = 1000, 2000 s/mm^2^(Experiment 2). Again, the MS fitting shows a good agreement with ground truth values, but a small systematic underestimation of the MD value is observed as MD of the tissue increases. This is most likely caused by Rician noise biases at the high-b-value of 2000 s/mm^2^.Figure 1C and Dshows the results obtained when tissue non-Gaussian effects are included in the simulated signals (Experiments 3 and 4, respectively). In both experiments, small biases can be observed for the MD and FW index estimates obtained with NLS fitting (MS), which increase with increasing MD. These biases were more noticeable when the fitted data included the high b-value of 2000 s/mm^2^(Experiment 4), especially for the FW index. Despite these biases, FA estimates remain close to the simulated ground truth. For the RGD fitting (SS), two very noticeable biases are observed across all experiments: firstly, the profile of MD estimates is remarkably flat and unable to track the increasing simulated MD values; secondly, the FA estimates also deviate significantly from ground truth, showing a systematic increase as the underlying MD of the tissue increases.

### 3.2 Comparing FWE estimates with “gold standard” using human data

In order to assess whether the same biases can be observed in human data, we used the extra dataset with lower b-values. In the simulation experiments, the results obtained from the MS FWE-DTI approach with the pair of b-values b = 300, 1000 s/mm^2^were closest to the simulated ground truth (Fig. 1A). Therefore, this method and pair of b-values will be used as the best approximation to the true underlying MD and FA estimates, or “gold standard,” for the human data analysis.

**Fig. 2.**
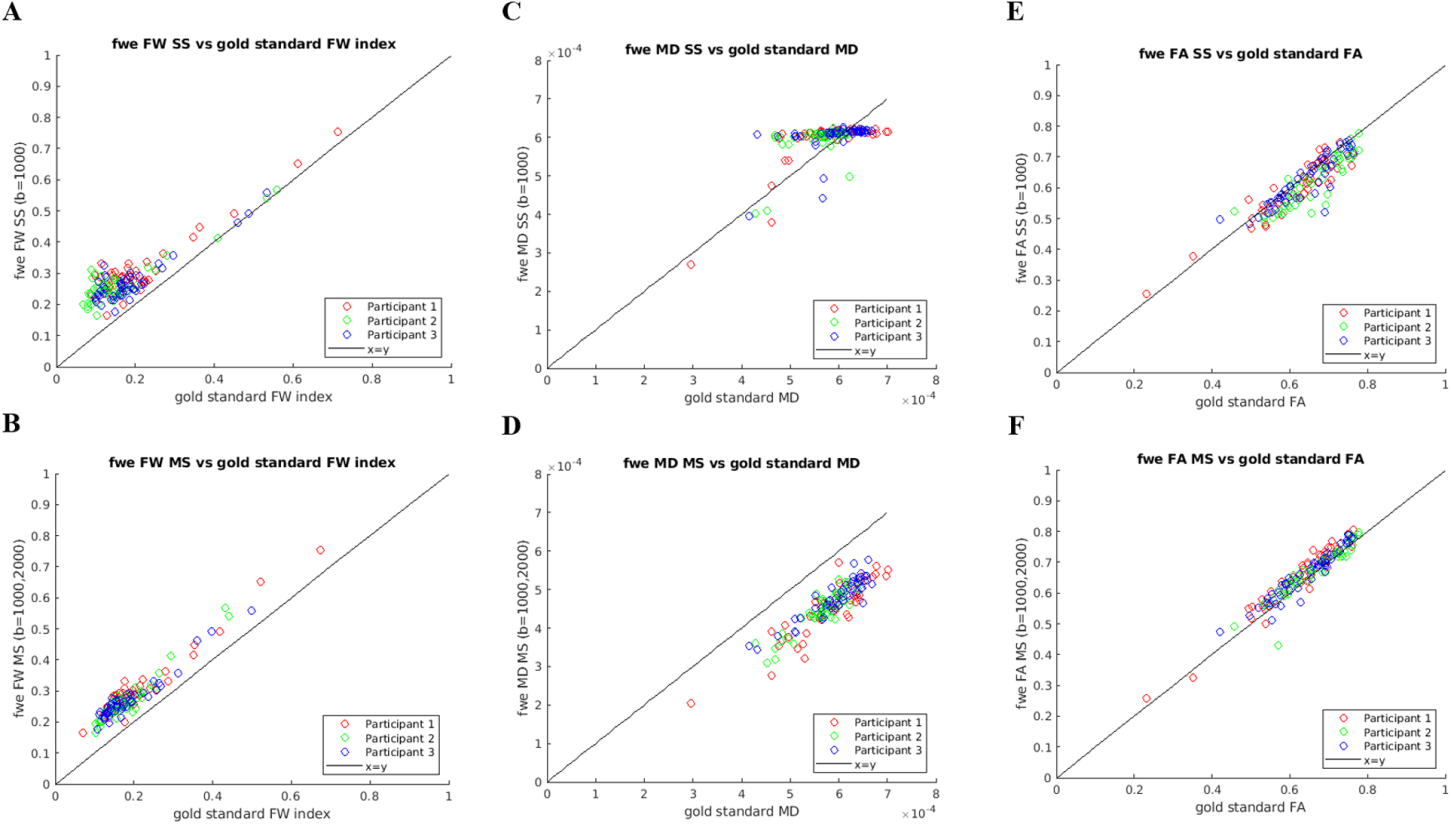
FWE-DTI estimates obtained from the extra dataset including a lower b-value shell, plotted against their corresponding “gold standard” (b = 300, 1000 s/mm^2^). Each point on these plots corresponds to the mean of the diffusion metric within a different JHU ROI. (A) fwe_FW_SS (RGD, b = 1000 s/mm^2^), (B) fwe_FW_MS (NLS, b = 1000, 2000 s/mm^2^), (C) fwe_MD_SS (RGD, b = 1000 s/mm^2^), (D) fwe_MD_MS (NLS, b = 1000, 2000 s/mm^2^), (E) fwe_FA_SS (RGD, b = 1000 s/mm^2^), and (F) fwe_FA_MS (NLS, b = 1000, 2000 s/mm^2^).

Figure 2shows the estimated FW index using both RGD on SS data (b = 1000 s/mm^2^, panel A) and NLS on MS data (b = 1000, 2000 s/mm^2^, panel B), against the “gold standard” FW index estimates (NLS MS fitted to b = 300, 1000 s/mm^2^data). Panels C and D show the equivalent plots for MD estimates, and panels E and F show the graphs for FA estimates.

Figure 2A and Bshows that both RGD SS and NLS MS fitting result in overestimation of the FW index relative to the “gold standard.”

The FWE MD estimates obtained from SS data clearly deviate from the “gold standard” MD, with the majority of the values clustering around the value set for MD’s tissue prior (0.6 μm^2^/ms or 0.6 × 10^-3^mm^2^/s). As predicted by the simulations inFigure 1D, the FWE MD estimates obtained from MS data with b = 1000, 2000 s/mm^2^are generally lower than the “gold standard” MD values. However, inFigure 2Dwe observe a linear pattern between fwe_MD_MS and the “gold standard” MD which is approximately parallel to the x = y line, which means that relative differences in MD between ROIs are preserved with the MS FWE-DTI fitting, despite the underestimation introduced by non-Gaussian diffusion effects and Rician noise biases.

Figure 2Esuggests that the FA estimates obtained with RGD SS are slightly underestimated relative to “gold standard” FA, while the NLS MS estimates are slightly higher than “gold standard” (Fig. 2F).

To further investigate whether the differences between the FWE-DTI MD and FA estimates and their corresponding “gold standard” are related to the underlying tissue MD, we also plotted those differences as a function of “gold standard” MD, and the results are shown inFigure 3.

**Fig. 3.**
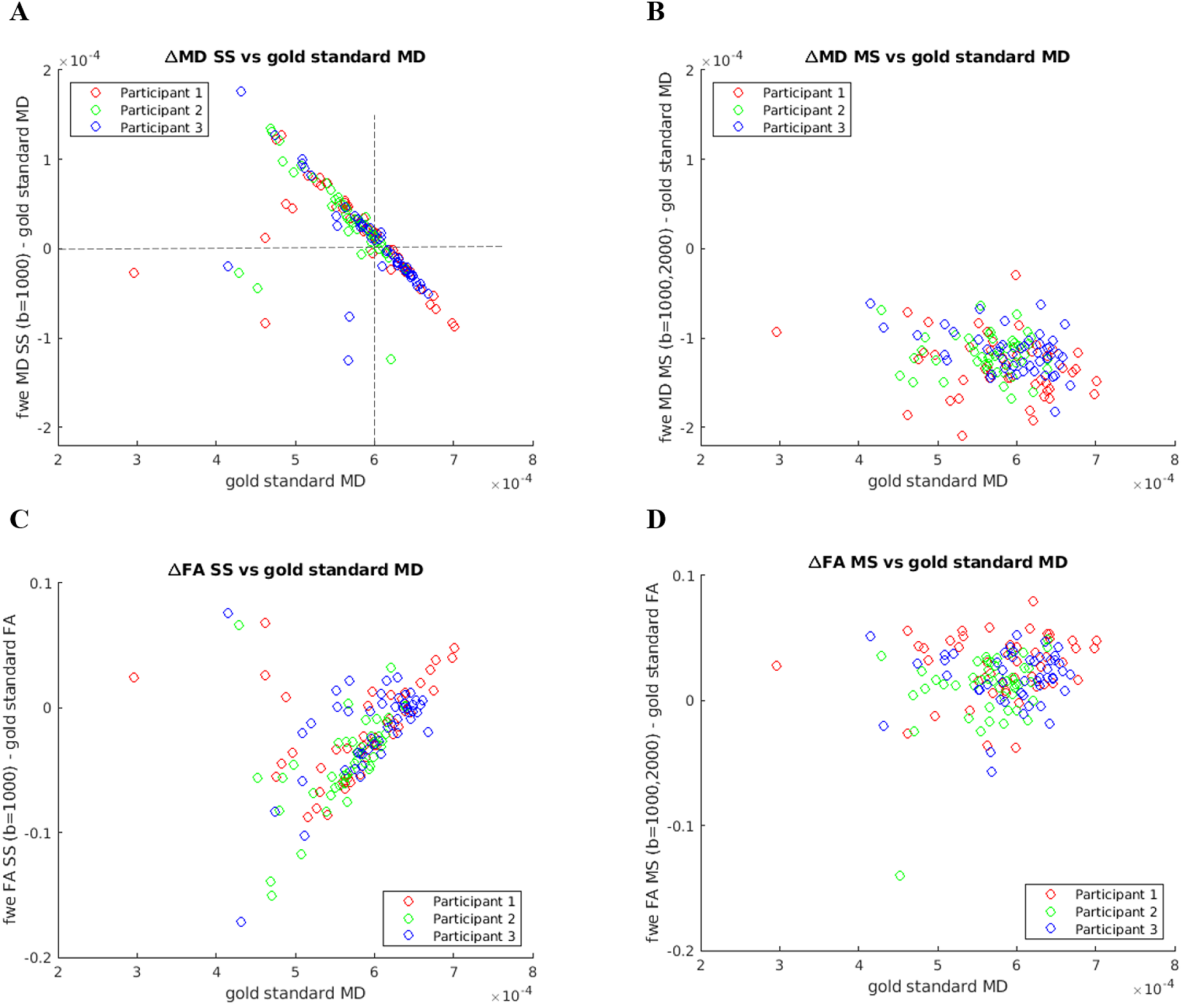
Difference between the estimated FWE-DTI MD and FA maps and their corresponding “gold standard” maps, shown as a function of “gold standard” MD. (A) ΔMD_SS (fwe_MD_SS – fwe_MD_GS), (B) ΔMD_MS (fwe_MD_MS – fwe_MD_GS), (C) ΔFA_SS (fwe_FA_SS – fwe_FA_GS), and (D) ΔFA_MS (fwe_FA_MS – fwe_FA_GS).

Figure 3Ashows that the difference between FWE MD obtained with RGD SS and “gold standard” MD decreases steeply with increasing “gold standard” MD. As predicted by the simulations (Fig. 1), this difference is positive for “gold standard” MD less than 0.6 × 10^-3^mm^2^/s and negative for “gold standard” MD greater than 0.6 × 10^-3^mm^2^/s (Fig. 3A, see dashed lines). The NLS method applied to the MS data including a b-value of 2000 s/mm^2^always underestimates the “gold standard” MD.Figure 3Bsuggests that there is also a slight decrease of the difference between fwe_MD_MS and “gold standard” MD, but this is much less steep than what is observed for fwe_MD_SS.Figure 3Cshows that the difference between fwe_FA_SS and “gold standard” FA generally increases with the underlying MD value of the tissue, which again matches the bias observed in simulations (Fig. 1). However, no such behaviour is observed with MS NLS fitting (Fig. 3D), which is also in agreement with the simulations, where FA estimates were always close to ground truth, despite the biases observed in MD and FW index (Fig. 1).

Versions ofFigures 2and3showing the data points colour coded by ROI are given inSupplementary Material, Appendix C.

### 3.3 TBSS analyses using the Cam-CAN dataset

**Fig. 4.**
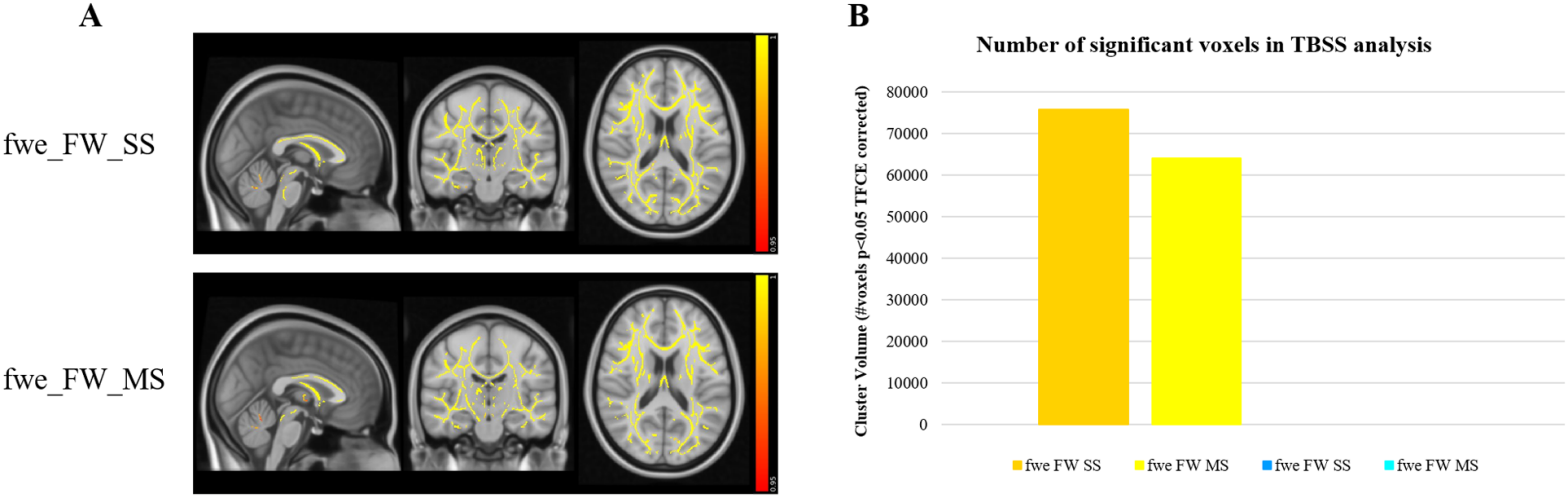
TBSS results showing widespread positive correlations between FW index and age. (A) Significance maps for both RGD SS FWE-DTI (fwe_FW_SS) and NLS MS FWE-DTI (fwe_FW_MS) and (B) number of significant voxels per fitting method.

**Fig. 5.**
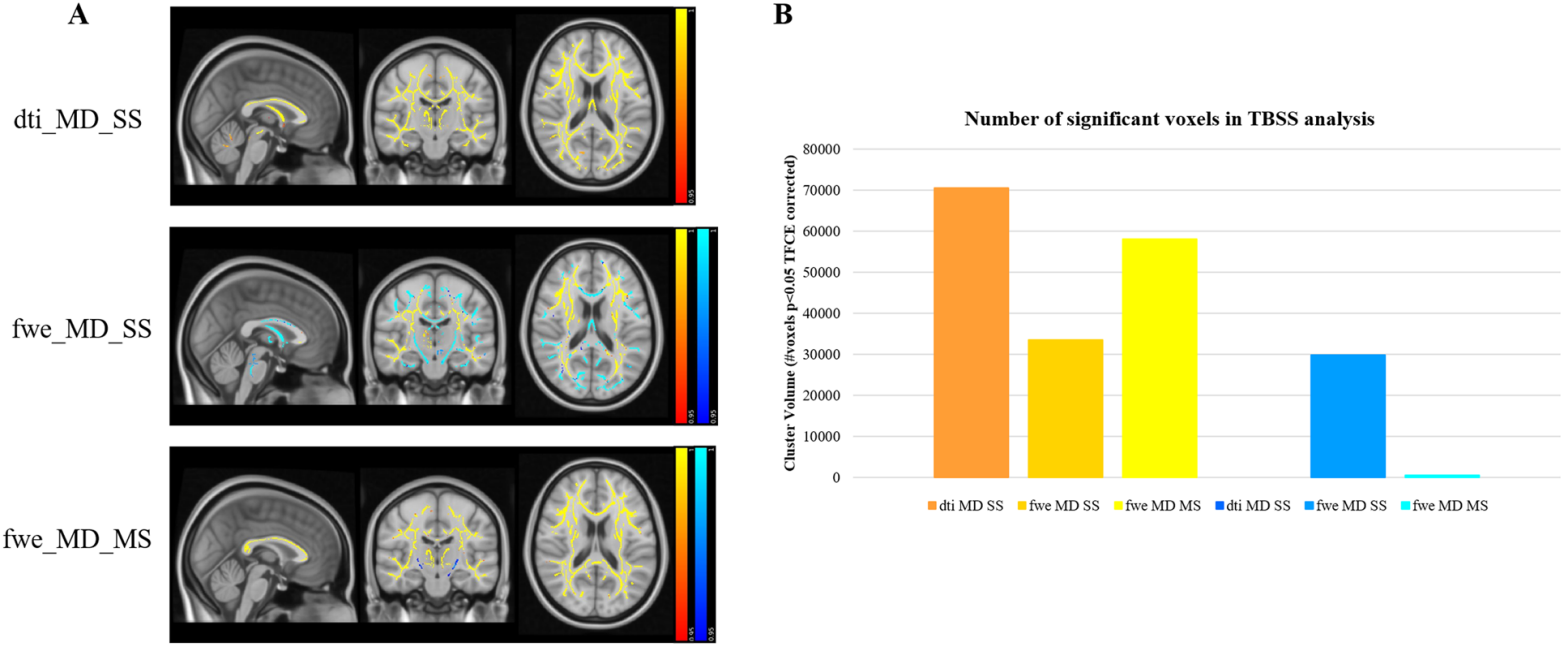
TBSS results showing widespread correlations between MD estimates and age. Positive correlations are shown in red/yellow and negative correlations are shown in blue. (A) Significance maps for DTI (dti_MD_SS), RGD SS FWE-DTI (fwe_MD_SS), and NLS MS (fwe_MD_MS) and (B) number of significant voxels per fitting method.

**Fig. 6.**
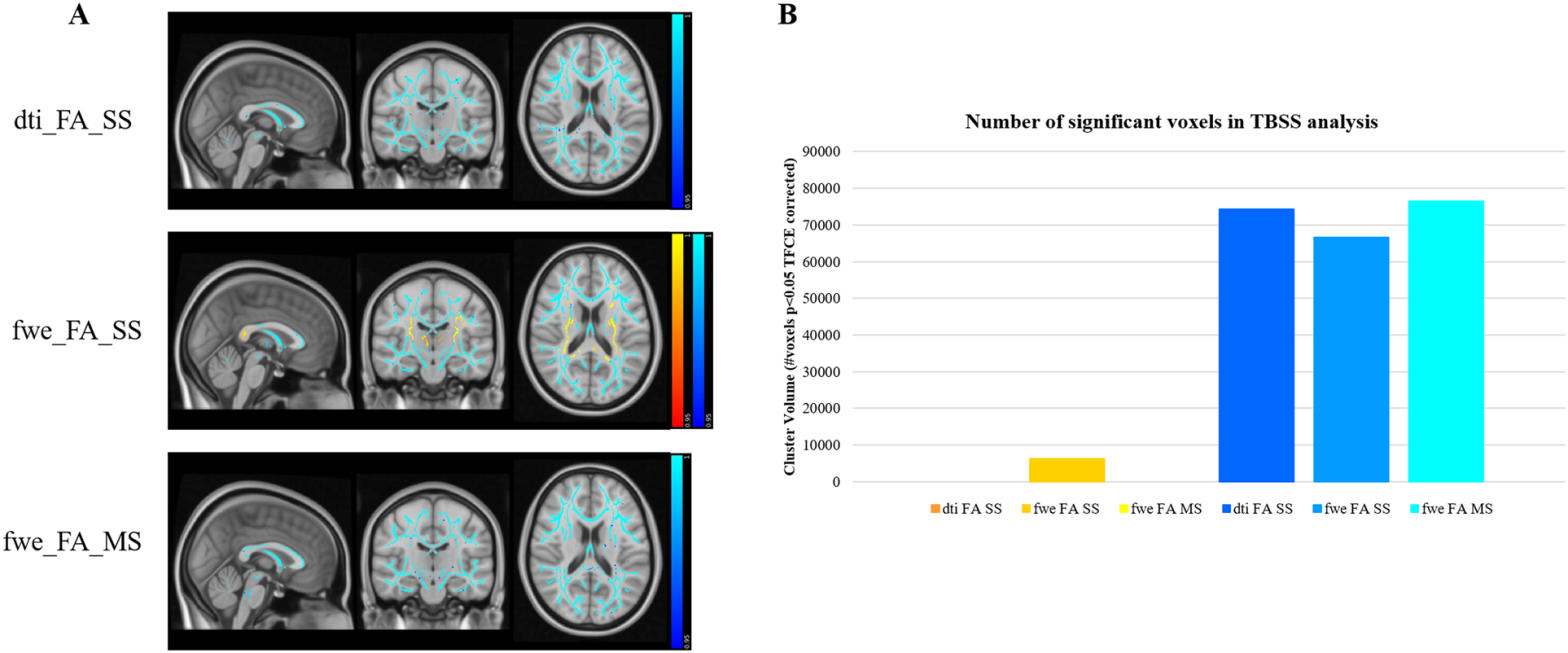
TBSS results showing widespread negative correlations between FA estimates and age, as well as some positive correlations for FWE-DTI FA maps only. Positive correlations are shown in red/yellow and negative correlations are shown in blue. (A) Significance maps for DTI (dti_FA_SS), RGD SS (fwe_FA_SS), and NLS MS FWE-DTI (fwe_FA_MS) and (B) number of significant voxels per fitting method.

Figures 4 to 6show the brain regions which were found to correlate with age for each of the eight studied diffusion metrics. Positive correlations with age are shown in the red/yellow scheme, while negative correlations are shown in blue.

The FW index shows widespread positive correlations with age, for both the SS and MS algorithms (Fig. 4A), but more significant voxels were identified with the SS RGD approach (Fig. 4B).

We found widespread positive correlations between MD and age, for all combinations of model fitting and dataset used (Fig. 5A). When FWE is performed, the number of significant voxels decreases, for both SS and MS approaches, but this effect is more pronounced for the RGD SS method (Fig. 5B). For the FWE methods, voxels showing a negative correlation between MD and age were also identified. For the RGD SS method, the number of such voxels was similar to the number of positive correlations for the same method, while for the NLS MS approach, the number of voxels showing negative correlations was very small (Fig. 5B).

The DTI-based FA metric shows strong negative correlations with age across the whole brain white matter (Fig. 6A). However, the FW-compensated maps produced strikingly different results depending on the method employed for FWE-DTI fitting: the maps obtained with the RGD SS method identified some brain areas with a strong positive association with age—contrasting the known prevalence of FA decline with age (Davis et al., 2009;Lebel et al., 2012;Sullivan et al., 2001)—while no such positive correlations were found with the MS NLS model (Fig. 6A).

### 3.4 ROI analysis using the Cam-CAN dataset

**Fig. 7.**
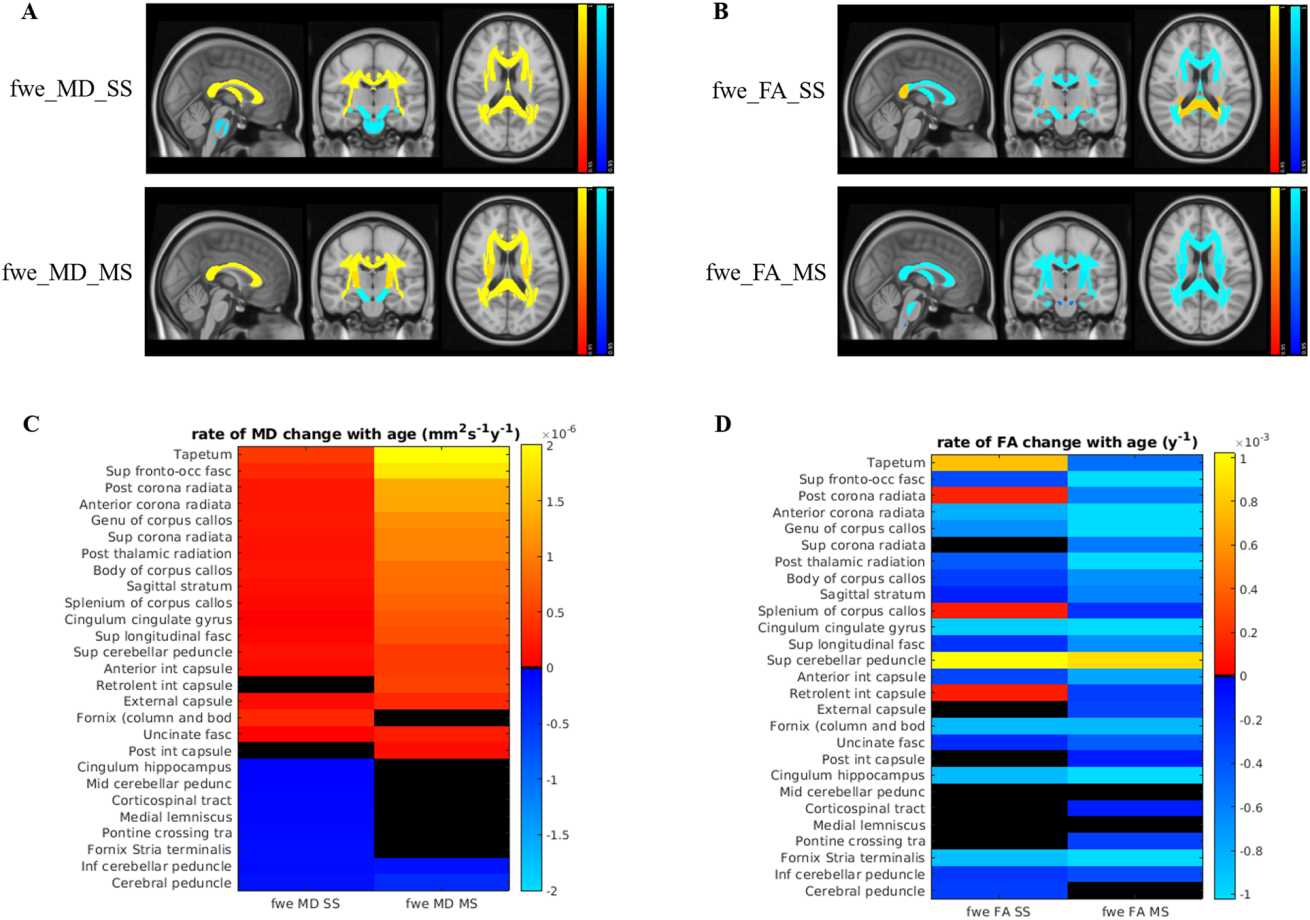
ROI-based linear modelling results, with positive correlations shown in red/yellow, while negative correlations are shown in blue. (A) Significance maps for MD correlations with age for RGD SS (fwe_MD_SS) and NLS MS FWE-DTI (fwe_MD_MS). To match TBSS results, the colour bars represent 1 – p-value. (B) Significance maps for FA correlations with age for the two FWE fitting methods (fwe_FA_SS and fwe_FA_MS). (C) Rate of MD change with age, as estimated by the linear models for each ROI and each FWE fitting method. Non-significant correlations with age are shown in black. (D) Rate of FA change with age, as estimated by the linear models for each ROI.

The results from the ROI-based analysis are summarised inFigure 7(scatter plots for each ROI, metric, and fitting method are given inSupplementary Material, Appendix D). The significant maps (Fig. 7A, B) correspond to 1 – p-value, in order to match the TBSS significance maps. Positive correlations are shown in red/yellow, while negative correlations are shown in blue.Figure 7C and Dshows the rates of change of MD and FA, respectively, for each ROI and each FWE-DTI fitting method, as estimated by linear models. Once again, positive rates are shown in red/yellow, while negative rates are shown in blue, and non-significant results are shown in black. The ROIs were ranked by mean rate of change of MD across both FWE-DTI methods. The results largely match the findings from TBSS. For MD, widespread positive correlations with age were found (Fig. 7A, C), as well as some negative correlations, especially for the RGD SS method. Overall, the rates of MD change with age were lower for the RGD SS method. For FA, positive correlations with age were found in five ROIs (Tapetum, Posterior Corona Radiata, Splenium, Superior Cerebellar Peduncle, and Retrolenticular part of the Internal Capsule) for the SS method, and for one ROI (Superior Cerebellar Peduncle) with MS FWE fitting. The latter is the most notable difference between the ROI and TBSS results, where no significant positive correlations between age and fwe_FA_MS were found.

Figure 7C and Dalso shows that for all ROIs where a positive correlation between fwe_FA_SS and age was found, a significant positive change in fwe_MD_MS was also present.

**Fig. 8.**
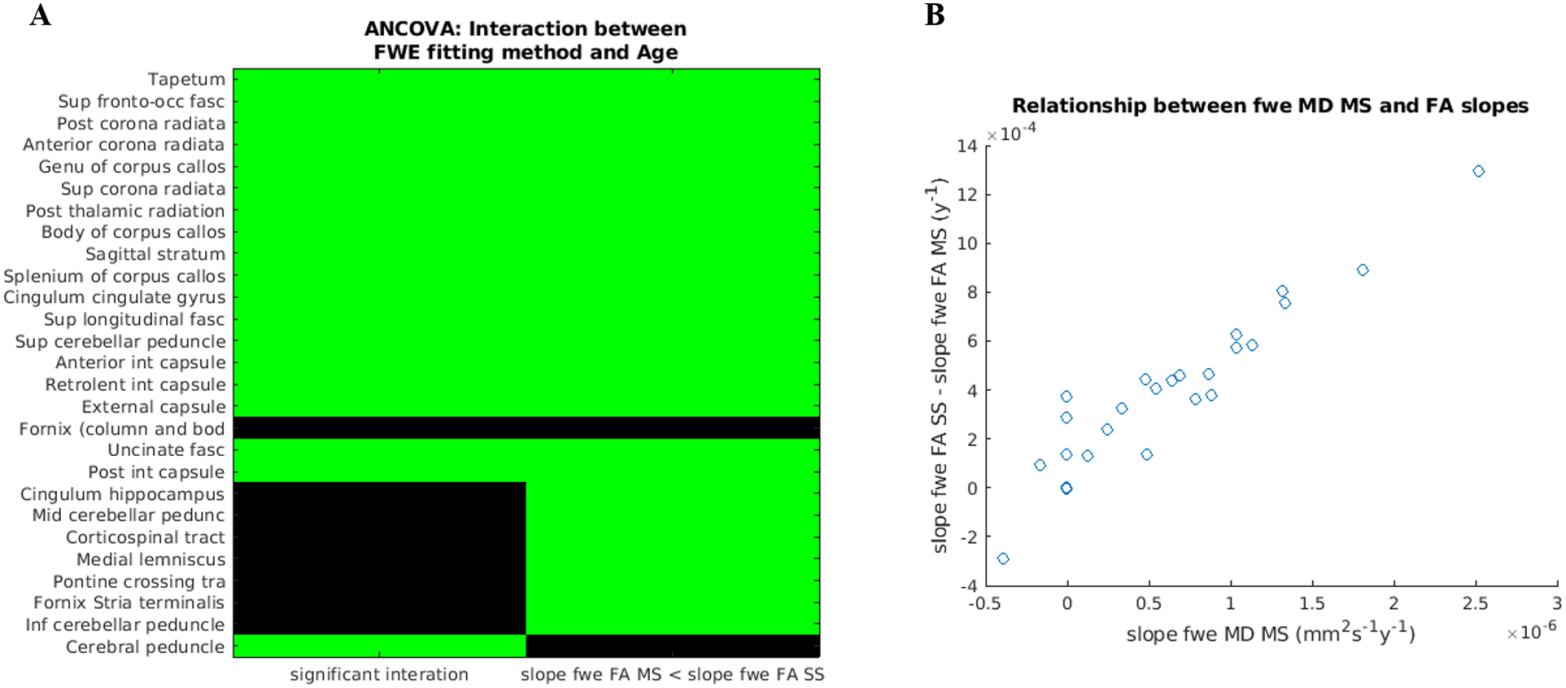
ANCOVA results per ROI. (A) The left column shows in green the ROIs where the interaction between age and FWE-DTI fitting method was found to be significant (p < 0.05, FDR corrected). The right column shows in green the ROIs where the slope estimate for the change of fwe_FA_MS with age was smaller than the one for fwe_FA_SS. (B) The difference between the FA slope estimates for each fitting method is plotted as a function of the rate of change in MD (as measured by fwe_MD_MS) for the same ROI. The ROIs are ordered to matchFigure 7.

The results from the ANCOVA are summarised inFigure 8A. The left column shows the ROIs where the interaction between age and FWE-DTI fitting method was found to be significant (p < 0.05, FDR corrected), and the right column highlights the ROIs where the slope estimate for the change of fwe_FA_MS with age was smaller than the one for fwe_FA_SS.Figure 8Bfurther shows that the difference between the FA slope estimates increases with the rate of change of MD (as measured by fwe_MD_MS) in the corresponding ROI.

## 4 Discussion

In the present study, we compared the performance of the RGD algorithm (Pasternak et al., 2009), with initialisation based on the tissue’s MD, proposed for FWE-DTI fitting using SS dMRI data, to a non-linear squares fitting approach using MS data, when applied to the study of healthy ageing in a cohort of 620 participants. The main goal was to determine whether both methods lead to comparable research findings, or whether the limitations of the RGD fitting method (as highlighted inGolub et al. (2021)) might lead to invalid or implausible observations.

Because the dataset selected for this analysis included a b-value of 2000 s/mm^2^, any results obtained with DTI modelling including this high b-value were likely to be affected by non-Gaussian diffusion effects and/or noise floor biases (Hoy et al., 2014;Neto Henriques et al., 2017). Therefore, we first needed to quantify the extent of this issue using both simulations and human data (the extra datasets with lower b-values). The simulation results were in line with previous reports (Neto Henriques, 2018;Neto Henriques et al., 2017), showing that MD and FW estimates obtained with the NLS algorithm applied to the pair of b-values b = 1000, 2000 s/mm^2^are biased relative to ground truth (Fig. 1B, D, green data). The MD values are slightly underestimated, and the magnitude of the undershoot increased for larger ground truth MD values and FW index (Fig. 1B, D, middle row). The FW estimates are generally higher than ground truth, and again the difference increased with increasing MD values (Fig. 1B, D, top row). These MD/FW biases shown inFigure 1Bcan be explained only by noise floor biases given that synthetic signals are generated according to the same FWE-DTI forward model. However, the more pronounced biases observed inFigure 1Dcan be explained by tissue non-Gaussian effects not included in simulation experiments 1 and 2. Interestingly, the FA estimates obtained with the b-value pair b = 1000, 2000 s/mm^2^do not appear to be noticeably affected by non-Gaussian and noise floor effects, with the exception of a residual FA overestimation for increasing MD ground truth values (Fig. 1D, bottom row).

The analysis of the extra datasets with lower b-values largely confirmed the observations from simulations: the fwe_FW_MS obtained with FWE-DTI applied to the b-value pair b = 1000, 2000 s/mm^2^is overestimated relative to “gold standard” (Fig. 2B), the fwe_MD_MS values are underestimated (Fig. 2D), while fwe_FA_MS is much closer to “gold standard” FA (Fig. 2F). The human data also suggest that non-Gaussian diffusion effects result in a slight overestimation of FA when the b = 2000 s/mm^2^shell is included, as also seen in simulations (Fig. 1D). Importantly, despite the over- and under-estimations described above, the distributions of points inFigure 2B, D, and Fare all roughly parallel to the line x = y, suggesting that relative differences between ROIs are largely preserved, despite the greater impact of tissue non-Gaussian diffusion and noise floor effects when data with a high b-value are used.

Careful comparison of the profiles for fwe_FW_SS (Fig. 2A) and fwe_FW_MS (Fig. 2B) suggests that the overestimation of the FW index is more pronounced for the RGD SS method, especially for lower values of “gold standard” FW index. This is again consistent with the simulation results, where the overestimation of the FW index was greatest for the lowest simulated FW index value and decreased as the ground truth FW index increased (Fig. 1A–D, from left to right plots).

The analysis of the extra b-value dataset further highlighted that when the RGD SS algorithm is applied to the b = 1000 s/mm^2^data, the fwe_MD_SS estimates are noticeably biased relative to “gold standard” MD. In particular, we observed clustering around the value set for the MD tissue prior ahead of the RGD fitting and the relative differences between ROIs are not preserved (a similar flattening effect is also observed in the simulations,Fig. 1).

Finally, the extra b-value dataset analysis also revealed that the overestimation of fwe_FA_SS relative to “gold standard” FA increased with the underlying MD of the tissue (Fig. 3A), while the same was not observed for fwe_FA_MS (Fig. 3B). The presence of tissue non-Gaussian diffusion/Rician noise bias effects introduced by the b = 2000 s/mm^2^shell does result in a small offset of fwe_FA_MS relative to “gold standard” FA, but the magnitude of this effect does not change with the underlying MD.

Next, we compared the ageing profiles predicted for each diffusion metric using the Cam-CAN dataset. Using TBSS, we found widespread positive correlations between age and the FW index, for both FWE-DTI fitting methods (Fig. 4), which is consistent with the known increase in CSF volume with age (Yamada et al., 2023). The RGD SS method resulted in a higher number of significant voxels. As discussed above, simulations and the extra b-value dataset analysis showed that both RGD SS and NLS MS including a high b-value of 2000 s/mm^2^overestimate the FW index, with the magnitude of this effect increasing with the underlying MD of the tissue. Therefore, it is likely that both approaches have overestimated the true number of brain voxels where the FW contamination increases significantly with age, with our results suggesting that this overestimation is more pronounced when RGD SS fitting is used. This conclusion is also consistent with the observations from simulations and the extra b-value dataset, which suggest that RGD SS fitting can lead to larger FW overestimation (see discussion above).

The analysis of the MD metrics revealed a large number of voxels where fwe_MD_SS correlated negatively with age (Fig. 5). This was also observed in the ROI analysis (Fig. 6A) where the fwe_MD_SS values were extracted in subject-specific space, and, therefore, this effect cannot be explained by any issues related to registration to a template or skeletonisation within TBSS. This effect has not been reported in previous studies of healthy ageing using RGD fitting applied to SS diffusion MRI data; however, it is in line with the work byChad et al. (2018)which reported a reduction in FWE-DTI MD effect size and reduced number of voxels showing positive correlations with age when compared with conventional DTI fitting. The large reduction in the rate of change of MD per year after FWE with RGD SS can be explained by the dramatic flattening of the fwe_MD_SS profile observed in the extra b-value dataset (Fig. 2C) and is a direct consequence of the tissue MD prior (MD_prior_= 0.6 × 10^-3^mm^2^/s) imposed as part of the RGD fitting algorithm. However,Chad et al. (2018)do not report any negative correlations with age, while we found them to be widespread in our data. The simulation results byGolub et al. (2021)(Fig. 1C) show that fwe_MD_SS values are underestimated for ground truth MD over 0.6 × 10^-3^mm^2^/s, and overestimated for ground truth MD below 0.6 × 10^-3^mm^2^/s. This can also be observed for the multi-b-value data (Fig. 2C), where the fwe_MD_SS estimates are above the x = y line for gold standard MD smaller than 0.6 × 10^-3^mm^2^/s and below the line for gold standard MD above 0.6 × 10^-3^mm^2^/s.Figure 3Ashows the difference between the fwe_MD_SS estimate and “gold standard” MD and it illustrates this issue clearly. Therefore, if we hypothesise that the ground truth tissue MD does increase with age (consistent with neurodegeneration processes such as demyelination and axonal degeneration previously reported in healthy ageing (Callaghan et al., 2014;Salvadores et al., 2017)), the underestimation introduced by the RGD SS fitting will lead to an overall decrease of the slope between fwe_MD_SS and age, which in some regions may result in an apparent negative relationship with age. Although not immediately visible to the naked eye inFigure 2D, asimilar effect is present for fwe_MD_MS (seeFig. 3B), and this was also seen in simulations (Fig. 1B, middle panel). The magnitude of this effect is much smaller than observed for fwe_MD_SS, but it can explain why some negative correlations between fwe_MD_MS are also observed for the NLS fitted data. It should be noted that this is likely a result of the non-Gaussian diffusion effects (and/or Rician noise bias) introduced by the 2000 s/mm^2^data, and not caused by the NLS MS fitting itself since the simulations for the b-value pair b = 300, 1000 s/mm^2^do not seem to be affected by this bias (Fig. 1A). The sample size included in our analysis was 620, compared with 212 inChad et al. (2018), which could explain why we find significant negative correlations with age, while none was reported byChad et al. (2018).

The TBSS results obtained with dti_FA_SS and fwe_FA_SS are in good agreement with those reported byChad et al. (2018): when conventional DTI was employed, we found only negative correlations between FA and age (Fig. 6A, top panel); after FW correction with RGD SS, we found a number of voxels with a strong positive association with age (Fig. 6A, middle panel). However, no such positive correlations were observed when the FW correction is performed with NLS MS (Fig. 6A, bottom panel).Chad et al. (2018)interpreted these positive correlations as related to selective degeneration of non-dominant tracts at fibre crossings. However, the replication of these observations when the RGD SS was used, but not with the well-posed NLS MS method, suggests that this apparent FA increase was most likely an artefact introduced by inappropriate SS data model fitting.

The ROI-based analysis allowed us to understand these discrepancies better. Overall, five ROIs showed a significant positive correlation between fwe_FA_SS and age (Fig. 7D): Tapetum, Posterior Corona Radiata, Splenium of the Corpus Callosum, Superior Cerebellar Peduncle, and Retrolenticular part of the Internal Capsule. These are in general agreement with the TBSS findings, but a perfect overlap between skeleton-based and ROI-based approaches cannot be achieved due to differences in pre-processing and statistical modelling. All the five ROIs showing a positive correlation between fwe_FA_SS and age were also found to show a significant fwe_MD_MS increase with age (Fig. 7C). This Figure also clearly shows the flattening effect of the RGD SS fitting on the estimated rates of MD change with age.

The ANCOVA allowed us to identify all ROIs where there is a significant interaction between age and the FWE fitting method for FA estimates, that is, whether the estimated rate of change of fwe_FA_MS with age was significantly different from the estimated rate for fwe_FA_SS.Figure 8Ashows that for the ROIs where there is a significant interaction, the slope estimate for fwe_FA_SS was always greater than the slope estimate for fwe_FA_MS when a significant increase of fwe_MD_MS was also found (Fig. 7C). The one exception to this was the Cerebral Peduncle, but in that case, the significant interaction was accompanied by a negative correlation between fwe_MD_MS and age.Figure 8Balso shows that the difference between the estimated rates of change for FA increased with the underlying MD change. All of these findings combined suggest that the fwe_FA_SS estimates are indeed tracking the underlying MD of the tissue (as measured by fwe_MD_MS), as already suggested by our simulations and analysis of the extra b-value dataset. In some ROIs, this effect was strong enough to be seen as a significant positive correlation between fwe_FA_SS and age, but all ROIs were affected in a consistent manner. The Cerebral Peduncle shows a negative correlation between fwe_MD_MS and age which, as discussed above, may be just a consequence of the biases introduced by tissue non-Gaussian diffusion effects in this brain ROI (Neto Henriques et al., 2023).

Figure 7Dshows that a significant positive correlation between fwe_FA_MS and age was found for the Superior Cerebellar Peduncle (SCP). This replicated the findings ofKuljiš et al. (2016), who also found a positive association between SCP microstructure and IQ. The authors hypothesised that these effects reflected the lifelong cognitive and motor training, which takes place in the cerebellum. Moreover, a recent study applying alternative models to Cam-CAN diffusion MRI data (Neto Henriques et al., 2023) revealed that FA increases in the SCP are associated with age-related alteration in fibre configuration (e.g., changes in the degree of fibre crossing/fanning/dispersion).

**Fig. 9.**
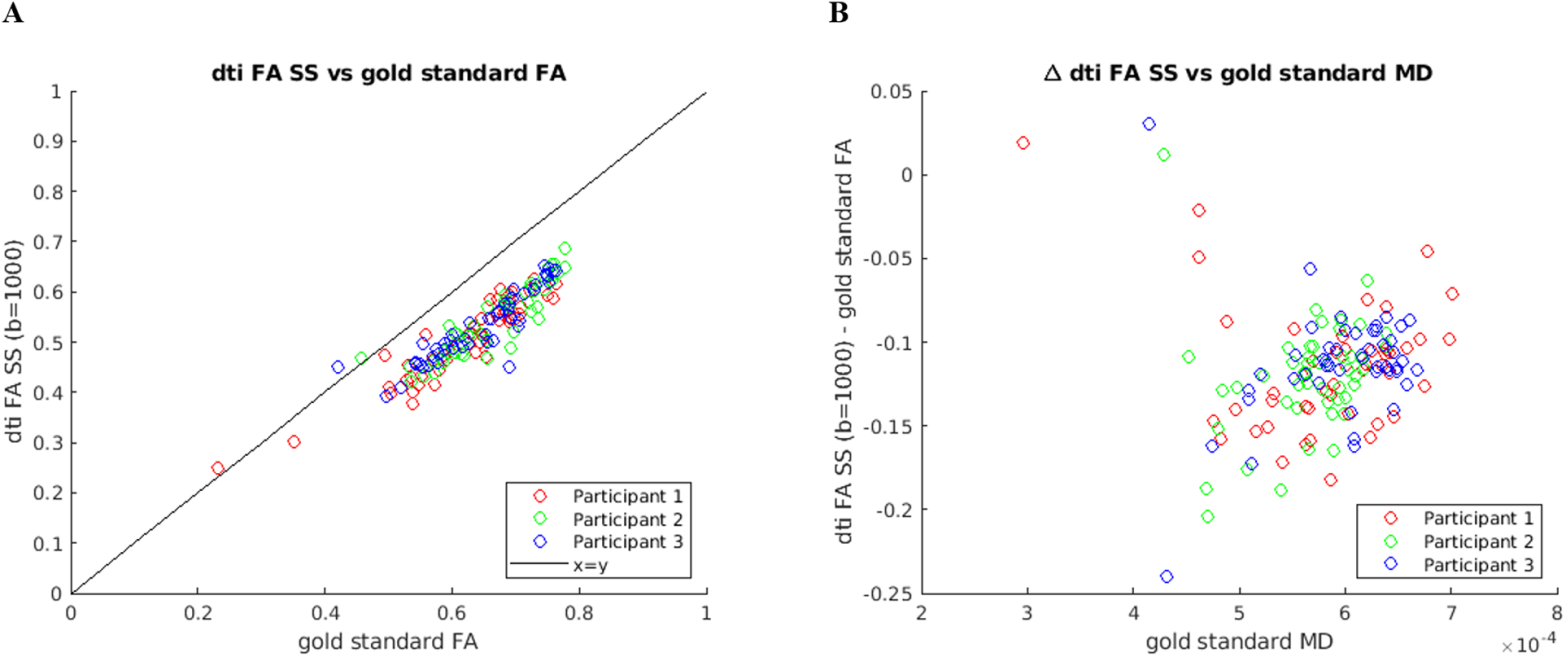
Illustration of the biases affecting standard DTI FA estimates. (A) dti_FA_SS plotted against “gold standard” FA, and (B) difference between the estimated dti_FA_SS and “gold standard” FA, shown as a function of “gold standard” MD.

Finally, it should be noted that positive correlations between FA and age, in similar brain regions to the ones observed with RGD SS fitting (Fig. 6A), have been previously reported on at least two occasions. The findings ofChad et al. (2018)have already been discussed above, and given the evidence provided in the present study, we believe these to be erroneous findings, explained by the biases introduced by the MD tissue prior used for RGD SS fitting. However,Miller et al. (2016)reported similar positive correlations between FA estimated by standard DTI (using data with a b-value of 1000 s/mm^2^) and age, on a much larger sample of 5000 UK Biobank participants. Those correlations were present for a TBSS analysis, but not for ROIs. The age range in the UK Biobank sample (40–69 years) is very different to the age range in the Cam-CAN dataset (18–88 years); therefore, in order to rule that out as a possible explanation for the discrepancy observed, we repeated our TBSS analyses for a subset of the Cam-CAN data matching the same age range as the UK Biobank (295 participants included). The results (seeSupplementary Material, Appendix B) were similar to the ones already reported for the full Cam-CAN dataset, with no positive correlations found for either dti_FA_SS or fwe_FA_MS. Given the much larger sample size included inMiller et al. (2016), it is possible that this is a true but small effect which cannot be detected with a more modest sample size. However, another possibility is that the positive correlations reported with the UK Biobank dataset and standard DTI FA are also erroneous. Using our extra b-value data, we can see that standard DTI underestimates the tissue FA value (Fig. 9A), and the magnitude of that underestimation again depends on the underlying MD of the tissues (Fig. 9B). Therefore, assuming that the MD of the tissue generally increases with age, it is possible that the increase in FA reported byMiller et al. (2016)is an artefact reflecting that increase in MD rather than true FA changes. The aim of the present study was to illustrate the biases introduced by RGD SS fitting, rather than making a novel contribution to the field of neurobiology of ageing. However, these discrepancies are clearly of interest and merit further investigations. A starting point for future work would be to perform FW correction on the UK Biobank dataset using NLS MS fitting.

The main limitation of the present study is the inability to fully rule out the presence of biases due to non-Gaussian diffusion effects on the FWE metrics using the b = 2000 s/mm^2^data. The simulations and extra-b-value data have allowed us to describe and quantify some of these issues, but we cannot make definitive statements about the negative correlations between fwe_MD_MS and age, for example. FWE modelling of more complex diffusion models (e.g., Diffusion Kurtosis Imaging (DKI), Neurite Orientation Dispersion and Density Imaging (NODDI)) was not performed because SS data only allow for DTI modelling, and the goal of this study was to compare SS with MS fitting. Moreover, the age-related trends of NODDI’s FW estimates using the Cam-CAN diffusion MRI data have been already explored in a recent study (Neto Henriques et al., 2023).

## 5 Conclusion

This study has shown that RGD fitting applied to SS dMRI data can lead to significant biases and erroneous findings. In particular, we have shown that these biases scale with the underlying MD of the tissues, leading to spurious correlations. The plausibility of the metrics generated with RGD fitting does not translate into accuracy and specificity, and, therefore, the findings reported in the literature using such methods should be interpreted with extreme care. Our results also replicated previous reports that NLS fitting including a high b-value results in biases for the corrected DTI metrics due to non-Gaussian diffusion effects. Therefore, future studies planning FWE analyses should include two b-values in a lower range to minimise FW estimation biases from non-Gaussian diffusion (e.g., b = 300, 1000 s/mm^2^as used for the extra b-value dataset).

## Supplementary Material

Supplementary Material

## Data Availability

The raw data in BIDS format are available on request from this website:https://camcan-archive.mrc-cbu.cam.ac.uk/dataaccess/. The code for the simulations is available herehttps://github.com/RafaelNH/fweDTI_SSvsMS, and the code/scripts used for data analysis are found herehttps://github.com/mmc43/fweDTI_SSvsMS.
