# Supplementary material for: The trouble with free-water elimination using single-shell diffusion MRI data: A case study in ageing
Source: Imaging Neurosci (Camb). 2024 Aug 1;2:imag-2-00252. doi: 10.1162/imag_a_00252 (PMC12272235; doi:10.1162/imag_a_00252)
Supplement: Supplementary Material [file imag_a_00252-supp.pdf]

Supplementary Material

The Trouble with Free-Water Elimination using Single-Shell Diffusion MRI Data: A Case-Study in Ageing

Marta M Correia[1,*], Rafael Neto Henriques[2], Marc Golub[3], Stefan Winzeck[4], Cam-CAN[5], Rita G Nunes[3]

[1]MRC Cognition and Brain Sciences Unit, University of Cambridge, Cambridge, United Kingdom

[2]Champalimaud Research, Champalimaud Centre for the Unknown, Lisbon, Portugal

[3]Institute for Systems and Robotics - Lisboa and Department of Bioengineering, Instituto Superior Técnico, Universidade de Lisboa, Lisbon, Portugal

[4]Division of Anaesthesia, Department of Medicine, University of Cambridge, Cambridge, United Kingdom

[5]Cambridge Centre for Ageing and Neuroscience (Cam-CAN), University of Cambridge, Cambridge, United Kingdom

***** corresponding author: marta.correia@mrc-cbu.cam.ac.uk

Appendix A – Simulation Details

Simulation experiments 1 and 2: synthetics signals were produced by directly using the free-water elimination (FWE)-DTI model (Pasternak et al., 2009; Pierpaoli & Jones, 2004):

$$S(b,\boldsymbol{n}) = S_o(F_w e^{-D_w b} + (1-F_w)e^{-b\boldsymbol{n}\boldsymbol{D_{tis}}\boldsymbol{n}^{\boldsymbol{T}}}) \quad (1)$$

where D_w is the isotropic free water diffusion coefficient (i.e. $D_w = 3$ μm²/ms), $\boldsymbol{D_{tis}}$ is the ground truth tissue anisotropic diffusion tensor, and F_w the ground truth apparent volume fraction (already taking into account the difference in T2-weighting in the signals for free water S_{water} and tissue components S_{tissue}), i.e.:

$$F_w = v_w S_{water}/(v_w S_{water} + (1-v_{water})S_{tissue}) \quad (2)$$

with v_w being the absolute free water volume fraction (Pasternak et al., 2009). Note that to introduce S_o dependency to the amount of free water S_{water} , it is set to 4.26×S_{tissue} assuming typical free water/tissue T2 values of 500ms/80ms (Golub et al., 2021; Piechnik et al., 2009; Wansapura et al., 1999), and free water/proton densities of 1/0.7 (Abbas et al., 2015; Golub et al., 2021), and TE=104ms. Similarly to the work by (Golub et al., 2021), simulations were repeated for different ground truth F_w values (sampled from 0 to 0.7), for different tissue mean diffusivities (MD_{tis}, here sampled between 0.2 to 1.1 μm²/ms), and for a fixed tissue fractional anisotropy FA_{tis} fixed to 0.7. From each combination of MD_{tis} and FA_{tis} ground truth values, ground truth diffusion tensors $\boldsymbol{D_{tis}}$ were constructed assuming radial symmetry, i.e.:

$$\lambda_1 = \frac{(1+2FA_{tis})}{\sqrt{3-3FA_{tis}^2}} MD_{tis}, \quad (3)$$

$$\lambda_2 = \lambda_3 = (3MD_{tis} - \lambda_1)/2. \quad (4)$$

Based on the above eigenvalues, $\boldsymbol{D_{tis}}$ are produced for over 10000 different instances: 10 different simulated diffusion tensor directions (to mitigate the dependency on a concrete diffusion gradient scheme in relation to the diffusion tensor main direction), repeated for 1000 instances and corrupted by Rician noise (nominal SNR=30 here defined as the signal-to-noise ratio computed using the ground truth signals S_{tissue} at b-value=0).

Simulation experiments 3 and 4: tissue non-Gaussian effects were represented by including independent signal attenuations for intra- and extra-cellular tissue compartments E_{intra} and

E_{extra}. The forward model for these simulations included, therefore, three different compartments:

$$S(b,\boldsymbol{n}) = S_o\{F_w e^{-D_w b} + (1 - F_w)[vE_{intra}(b,\boldsymbol{n}) + (1 - v)E_{extra}(b,\boldsymbol{n})]\} \quad (5)$$

where v is the apparent relative volume fraction between the intra and extra-cellular space. E_{intra} and E_{extra} were modelled according to the Neurite Orientation Dispersion and Density model (NODDI, (Zhang et al., 2012)) without fixing the intrinsic axonal diffusivity d:

$$E_{intra}(b,\boldsymbol{n}) = \int f(\boldsymbol{u}) \exp[-bd(\boldsymbol{n}^T\boldsymbol{u})^2]d\Omega_{\boldsymbol{u}} \quad (6)$$

and

$$E_{extra} = \exp\{-b\boldsymbol{n}^T[\int f(\boldsymbol{n})\, \boldsymbol{D}_{extra}(\boldsymbol{u})d\Omega_{\boldsymbol{u}}]\boldsymbol{n}\} \quad (7)$$

where $f(\boldsymbol{n}) = {}_1F_1\left(\frac{1}{2},\frac{3}{2},k\right)^{-1} \exp[-k(\boldsymbol{\mu}^T\boldsymbol{n})^2]$ is the fibre orientation distribution described by a Watson distribution with main direction $\boldsymbol{\mu}$ and concentration parameter k, ${}_1F_1$ is the confluent hypergeometric function, $\boldsymbol{D}_{extra}$ is an axial symmetric tensor aligned to vector $\boldsymbol{u}$ and with axial and radial diffusivities equal to d and $d(1-v)$ respectively. The NODDI model is selected for simulation experiments 3 and 4 since its independent parameters can be adjusted according to the desired apparent tissue diffusion tensor parameters MD_{tis} and FA_{tis} (Edwards et al., 2017; Jespersen et al., 2012). For this, we fixed the apparent relative volume fraction v to 0.7, so that different intrinsic diffusivity d and dispersion measure τ (which is related to k, (Jespersen et al., 2012; Zhang et al., 2012)) can be calculated for the different desired MD_{tis} (sampled from 0.2 to 1.1 μm^2/ms) and FA_{tis} (set to 0.7) values:

$$d = cMD_{tis} \quad (8)$$

and

$$\tau = \frac{1}{3}\left(1 + \frac{4}{c-1}\frac{FA_{tis}}{\sqrt{3-2FA_{tis}^2}}\right) \quad (9)$$

with $c = 3/(2(1-v)^2 + 1)$.

As for the previous experiments, simulated signals for experiments 3 and 4 are produced for over 10000 different instances: 10 different main orientations for the Watson orientation distribution function (to remove the dependency on a concrete diffusion gradient scheme in relation to the diffusion tensor main direction) repeated for 1000 instances corrupted by Rician

noise (nominal SNR=30, defined as the signal-to-noise ratio computed using the ground truth signals $S_{tissue}(0) = vE_{intra}(0) + (1 - v)E_{extra}(0)$).

<u>Synthetic signals post-processing:</u> For all experiment types, FA, MD and FW estimates for the multi-shell approach are obtained by fitting the multi-shell NLS algorithm (Neto Henriques et al., 2017) to the synthetic signals for all b-values. On the other hand, FA, MD and FW estimates for the single-shell approach are obtained by fitting the FWE-DTI MD-INI approach described by (Golub et al., 2021) to the synthetic signals for b-values = 0 and 1000s/mm^2. Note that spatial regularisation using the Laplace-Beltrami operator is not applied for the FWE-DTI SS estimates in synthetic data since simulations are produced for single voxels. FWE-DTI SS estimates are, however, representative of the FWE-DTI SS estimates obtained for the in vivo data as (Golub et al., 2021) showed that the accuracy of this technique is determined by its initialisation routine.

The median and interquartile ranges for the SS and MS FWE-DTI estimates (FA, MD, and FW) are then plotted as a function of the simulated ground-truth values of MD_{tis} and FW. In this study, we define the ground truth MD_{tis} and FA_{tis} estimates as the expected apparent tissue MD and FA values computed by standard DTI when free water is absent. Given this, the exact MD_{tis} and FA_{tis} ground truth values for simulations 3 and 4 are readjusted to the MD and FA values obtained by fitting DTI to the noise-free tissue signal attenuations (i.e., $S_{tissue}(b, \boldsymbol{n}) = vE_{intra}(b, \boldsymbol{n}) + (1 - v)E_{extra}(b, \boldsymbol{n})$).

<u>Multi-voxel phantom simulations</u>: to confirm that the determinant factor for SS FWE-DTI fit accuracy is its initialisation method, all simulations above are repeated for multi-voxel phantoms in which SS FWE-DTI estimates are extracted using the RGD approach, including Laplace-Beltrami spatial regularisation (supplementary Figure 1). For these simulations, 9261 replicas of the noise free diffusion-weighted signals for each of the above single voxel simulation are regrouped into phantoms with 21× 21×21 voxels. For computational speed, SS and MS FWE-DTI estimates from these phantoms are extracted directly from noise free signals (i.e. contrary to Figure 1, noise effects are not considered in supplementary Figure 1). Final FW, FA, and MD estimates for the multi-voxel estimates are extracted from the phantom’s middle voxel as no spatial estimation variation is observed for these noise-free synthetic phantoms.

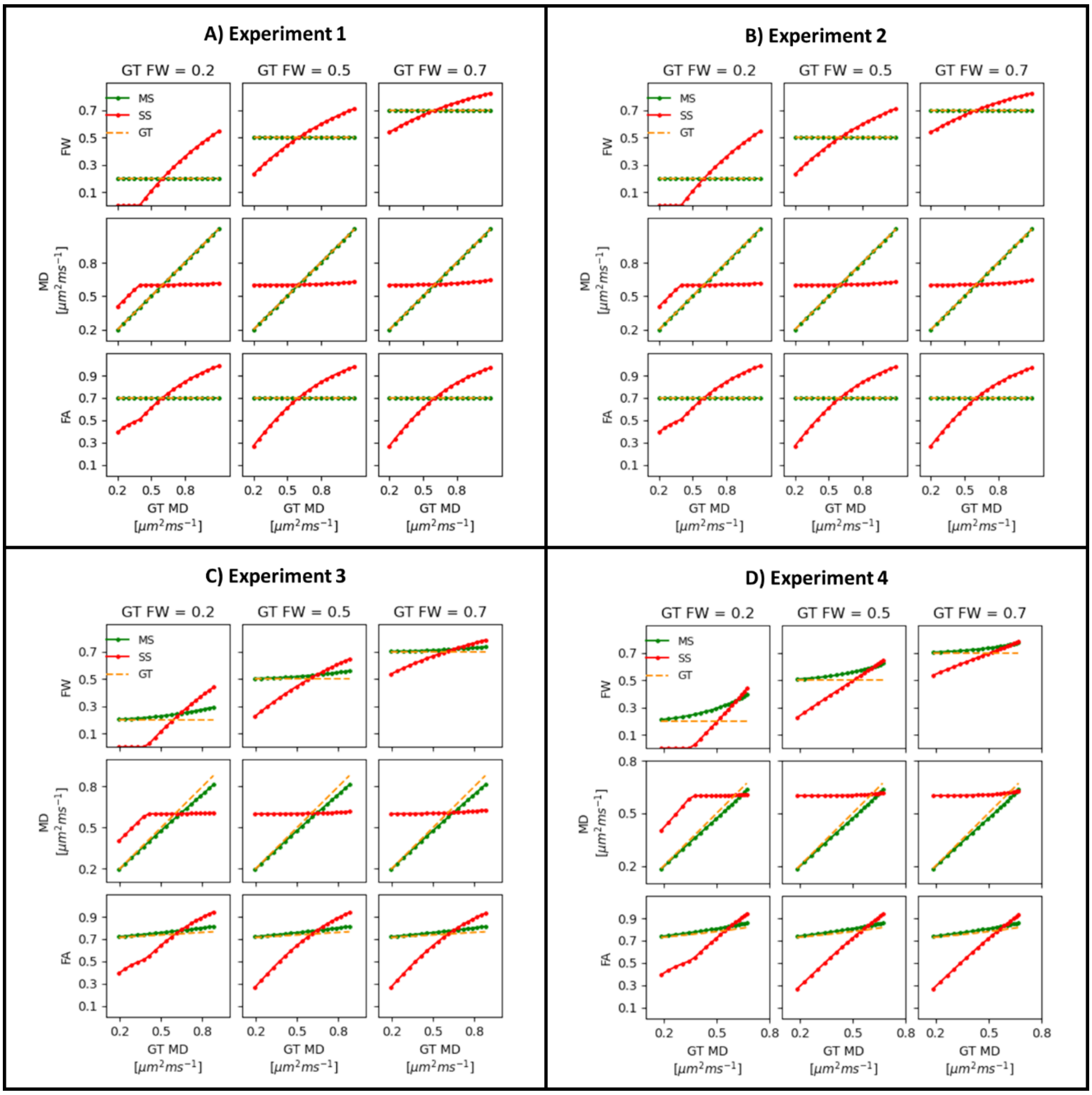

Supplementary Figure 1 - Single-shell (SS) and multi-shell (MS) FWE-DTI estimated from multi-voxel simulates for four simulation experiments considering different acquisition parameters and distinct effects of water diffusion in biological systems: A) synthetic signals for b=300, 1000 s/mm^2 ignoring non-Gaussian diffusion effects; B) synthetic signals for b=1000, 2000 s/mm^2 ignoring non-Gaussian diffusion effects; C) synthetic signals for b=300, 1000 s/mm^2 and considering non-Gaussian diffusion effects; D) synthetic signals for b=1000, 2000 s/mm^2 considering non-Gaussian diffusion effects. For each panel, FW, MD, and FA estimates are plotted as a function of the ground truth MD (1st, 2nd and 3rd rows of plots, respectively) for different ground truth free water contaminations (FW= 0.2, 0.5, 07 for columns of plots, respectively).

In general, supplementary Figure 1 shows the same biases reported in Figure 1, i.e.: 1) the profile of MD estimates is remarkably flat and unable to track the increasing simulated MD values; and 2) the FA estimates showing a systematic increase as the underlying MD of the tissue increases. The only difference in supplementary Figure 1 is that negative free water

estimates reported in Figure 1 are now truncated to zero by our Laplace-Beltrami regularisation code implementations. In this case, SS FWE-DTI fit retrieves the same MD and FA estimates than standard diffusion tensor fitting.

All code used to generate and analyse all single- and multi-voxel simulations are available at (https://github.com/RafaelNH/fweDTI_SSvsMS).

Appendix B – TBSS results for subset of CamCAN with age range 40-69 years (matching Miller et al., 2016)

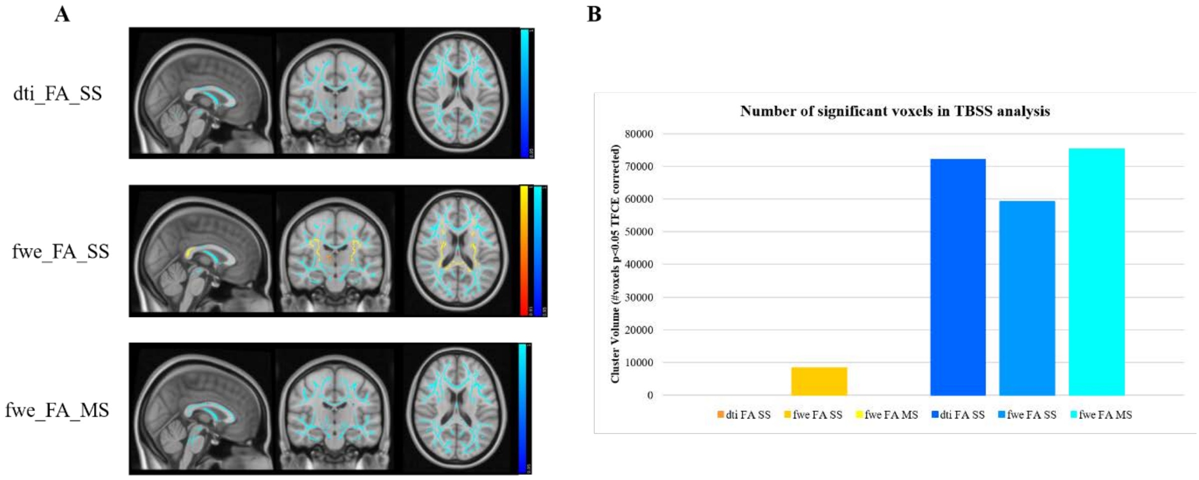

Supplementary Figure 2 – TBSS results for a subset of CamCAN participants (age range: 40-69 years). The results largely match the ones obtained with the full dataset (age range: 18-88 years). Panel A shows widespread negative correlations between FA estimates and age, as well as some positive correlations for FWE FA maps only. Positive correlations are shown in red/yellow and negative correlations are shown in blue. A) Significance maps for DTI (dti_FA_SS), RGD single-shell FWE (fwe_FA_SS) and NLS multi-shell (fwe_FA_MS) and B) number of significant voxels per fitting method.

Appendix C – Results for the dataset with a lower b-value shell, colour coded by ROI

The Figures below are identical to Figures 2 and 3 included in the main manuscript, but here the different ROIs have been colour coded. It can be seen that ROIs that are in close proximity in the brain also tend to cluster together, in particular left (L) and right (R) sub-divisions of the same ROI.

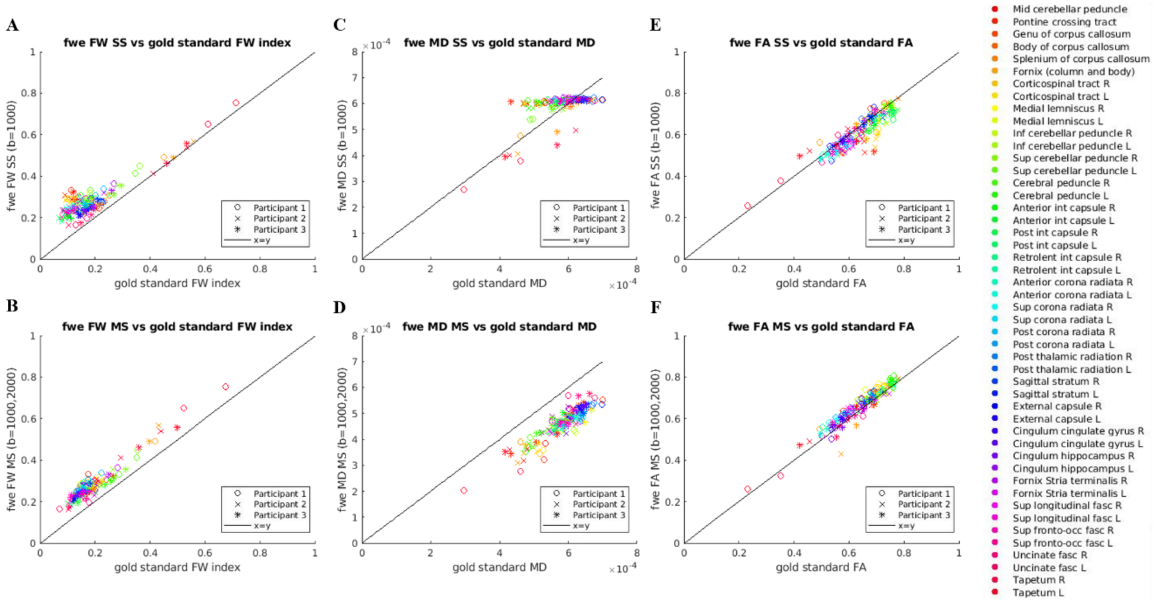

Supplementary Figure 3 – FWE-DTI estimates obtained from the extra dataset including a lower b-value shell, plotted against their corresponding ‘gold standard’ (b=300, 1000 s/mm^2). Each point on these plots corresponds to the mean of the diffusion metric within a different JHU ROI. A) fwe_FW_SS (RGD, b=1000 s/mm^2), B) fwe_FW_MS (NLS, b=1000, 2000 s/mm^2), C) fwe_MD_SS (RGD, b=1000 s/mm^2), D) fwe_MD_MS (NLS, b=1000, 2000 s/mm^2), E) fwe_FA_SS (RGD, b=1000 s/mm^2) and F) fwe_FA_MS (NLS, b=1000, 2000 s/mm^2). The data is the same as shown in Figure 2, except here ROIs have been colour coded.

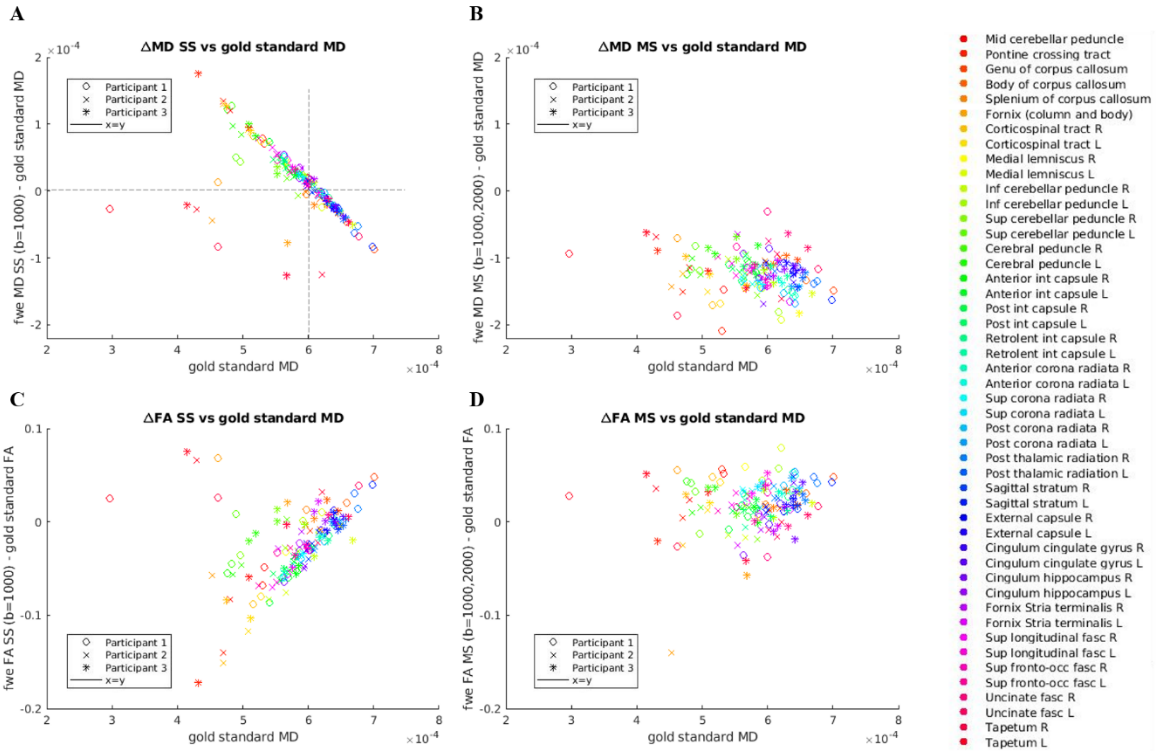

Supplementary Figure 4 – Difference between the estimated FWE-DTI MD and FA maps and their corresponding ‘gold standard’ maps, shown as a function of ‘gold standard’ MD. A) ΔMD_SS (fwe_MD_SS – fwe_MD_GS), B) ΔMD_MS (fwe_MD_MS – fwe_MD_GS), C) ΔFA_SS (fwe_FA_SS – fwe_FA_GS), and D) ΔFA_MS (fwe_FA_MS – fwe_FA_GS). The data is the same as shown in Figure 3, except here ROIs have been colour coded.

Appendix D – Scatter plots for FA and MD as a function of age for all ROIs

The Figures below show scatter plots between age and the diffusion tensor metrics estimated using the NLS MS algorithm, as well as RGD SS, for all the ROIs included in this study. The ROIs are presented in the same order as in Figures 7 and 8 (i.e., ranked in descending order of mean rate of change of MD across both FWE-DTI fitting methods).

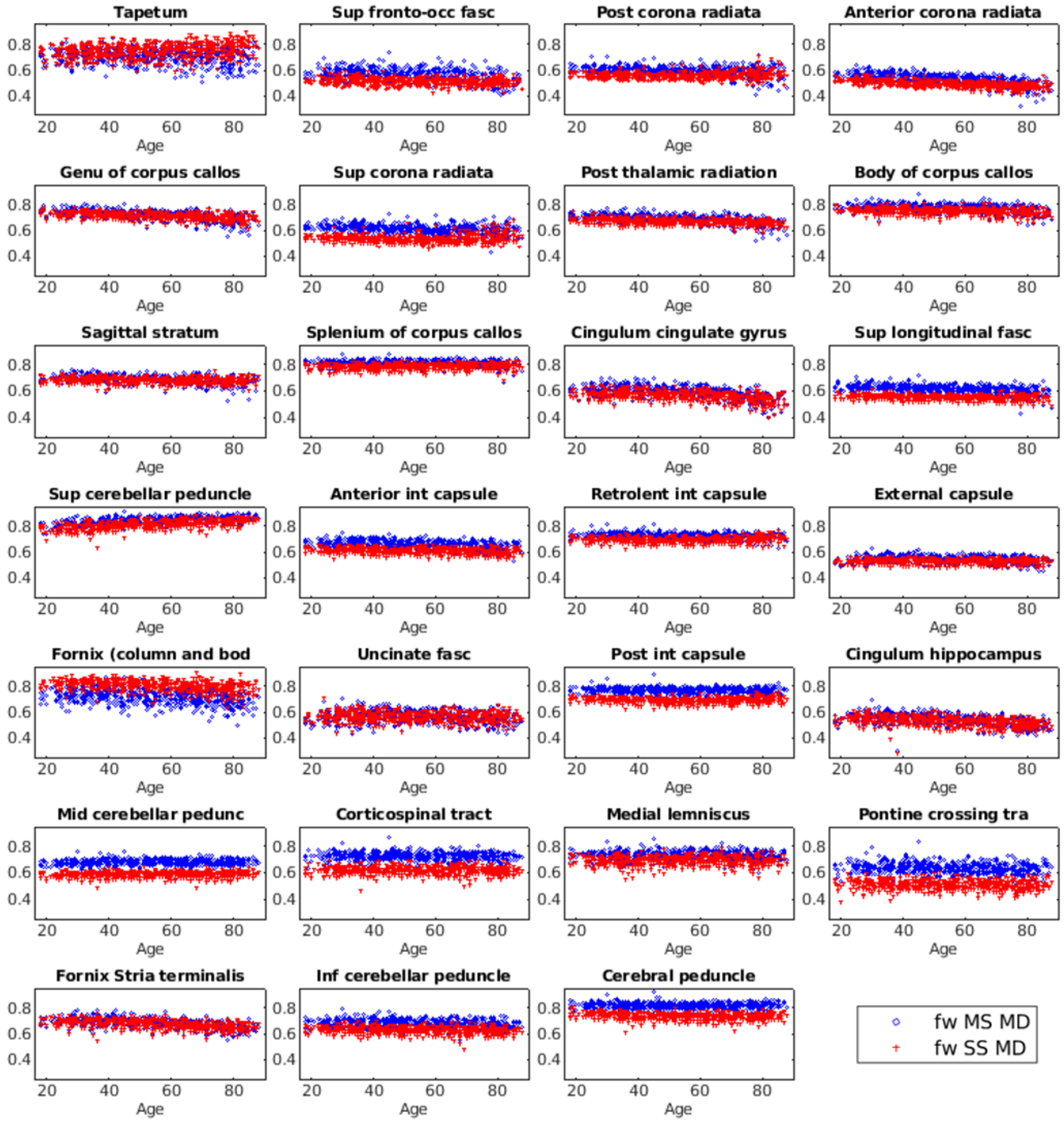

Supplementary Figure 5 – Scatter plots between FA and age for all ROIs. Data for fw_MS_FA are shown in blue, and data for fw_SS_FA are shown in red.

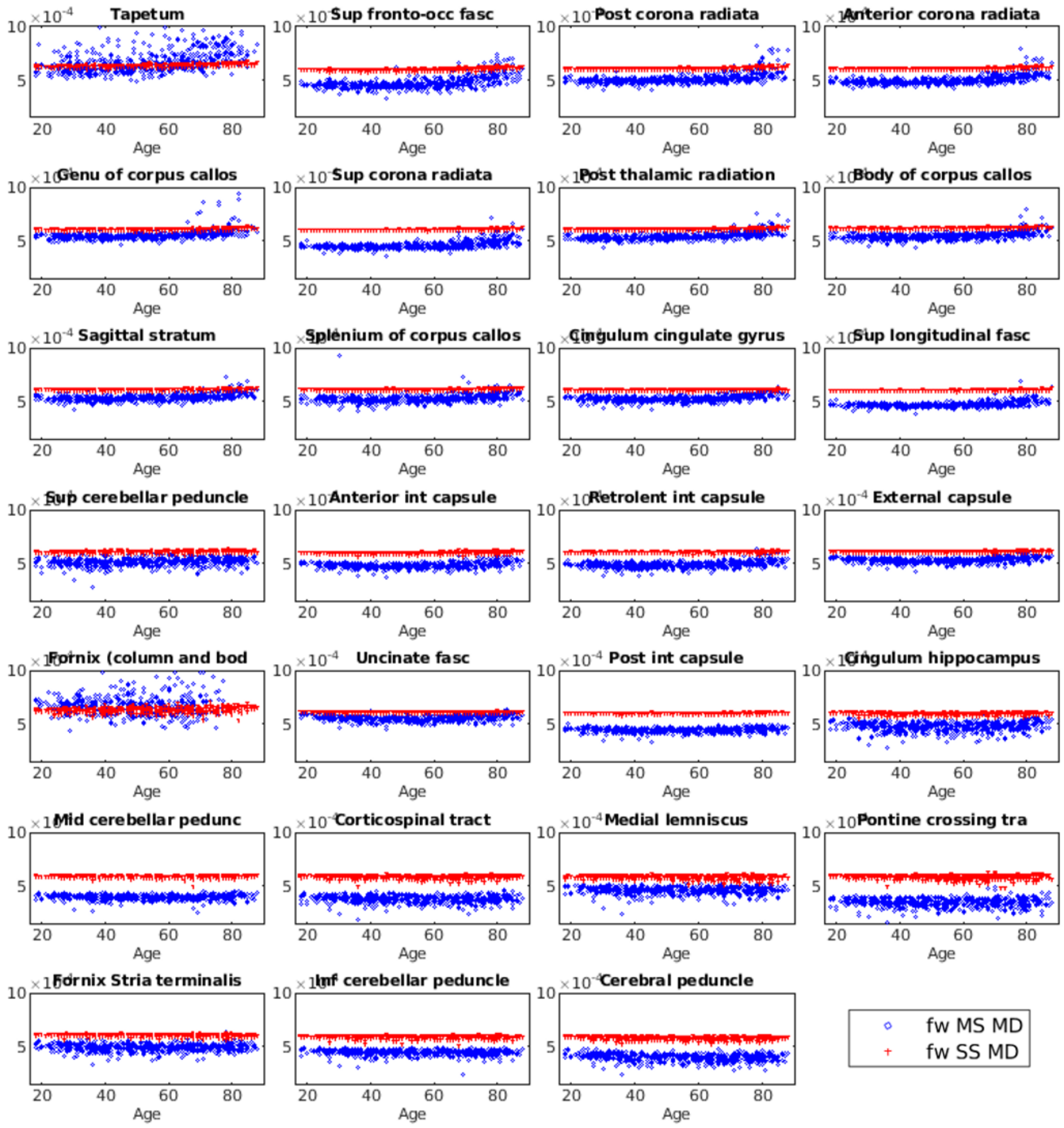

Supplementary Figure 6 – Scatter plots between MD and age for all ROIs. Data for fw_MS_MD are shown in blue, and data for fw_SS_MD are shown in red.

References

Abbas, Z., Gras, V., Möllenhoff, K., Oros-Peusquens, A. M., & Shah, N. J. (2015). Quantitative water content mapping at clinically relevant field strengths: A comparative study at 1.5 T and 3 T. *NeuroImage*, *106*, 404–413. https://doi.org/10.1016/J.NEUROIMAGE.2014.11.017

Edwards, L. J., Pine, K. J., Ellerbrock, I., Weiskopf, N., & Mohammadi, S. (2017). NODDI-DTI: Estimating neurite orientation and dispersion parameters from a diffusion tensor in healthy white matter. *Frontiers in Neuroscience*, *11*(DEC), 286694. https://doi.org/10.3389/FNINS.2017.00720/BIBTEX

Golub, M., Neto Henriques, R., & Gouveia Nunes, R. (2021). Free-water DTI estimates from single b-value data might seem plausible but must be interpreted with care. *Magnetic Resonance in Medicine*, *85*(5), 2537–2551. https://doi.org/10.1002/MRM.28599

Jespersen, S. N., Leigland, L. A., Cornea, A., & Kroenke, C. D. (2012). Determination of axonal and dendritic orientation distributions within the developing cerebral cortex by diffusion tensor imaging. *IEEE Transactions on Medical Imaging*, *31*(1), 16–32. https://doi.org/10.1109/TMI.2011.2162099

Neto Henriques, R., Rokem, A., Garyfallidis, E., St-Jean, S., Thomas Peterson, E., & Morgado Correia, M. (2017). *(PDF) [Re] Optimization of a free water elimination two-compartment model for diffusion tensor imaging*. https://www.researchgate.net/publication/315823060_Re_Optimization_of_a_free_water_elimination_two-_compartment_model_for_diffusion_tensor_imaging

Pasternak, O., Sochen, N., Gur, Y., Intrator, N., & Assaf, Y. (2009). Free water elimination and mapping from diffusion MRI. *Magnetic Resonance in Medicine*, *62*(3), 717–730. https://doi.org/10.1002/MRM.22055

Piechnik, S. K., Evans, J., Bary, L. H., Wise, R. G., & Jezzard, P. (2009). *Functional Changes in CSF Volume Estimated Using Measurement of Water T 2 Relaxation*. https://doi.org/10.1002/mrm.21897

Pierpaoli, C., & Jones, D. K. (2004). Removing CSF Contamination in Brain DT-MRIs by Using a Two-Compartment Tensor Model. *Proc. Intl. Soc. Mag. Reson. Med*, *11*.

Wansapura, J. P., Holland, S. K., Dunn, R. S., & Ball, W. S. (n.d.). *NMR Relaxation Times in the Human Brain at 3.0 Tesla*. https://doi.org/10.1002/(SICI)1522-2586(199904)9:4

Zhang, H., Schneider, T., Wheeler-Kingshott, C. A., & Alexander, D. C. (2012). NODDI: practical in vivo neurite orientation dispersion and density imaging of the human brain. *NeuroImage*, *61*(4), 1000–1016. https://doi.org/10.1016/J.NEUROIMAGE.2012.03.072
